# Longitudinal assessment of chorea in Huntington’s disease using digital passive monitoring

**DOI:** 10.1038/s41746-026-02661-y

**Published:** 2026-04-25

**Authors:** Claire Lugrin, Lidia Alecci, Cedric Simillion, Ekaterina Volkova-Volkmar, Louis-Solal Giboin, Fiona C. Kinsella, Peter McColgan, Edward J. Wild, Jonas Dorn

**Affiliations:** 1 Computational Sciences Center of Excellence, F. Hoffmann-La Roche Ltd, Basel, Switzerland; 2 Università della Svizzera italiana (USI), Lugano, Switzerland; 3 Huntington’s Disease Centre, UCL Queen Square Institute of Neurology, University College London, London, UK; 4 Roche Products Ltd, Welwyn Garden City, UK

**Keywords:** Diseases, Health care, Medical research, Neurology

## Abstract

Chorea is a hallmark of Huntington’s disease (HD), yet its clinical assessment is of limited reliability, impairing our ability to sensitively evaluate its progression. We evaluated passive monitoring for estimating the two-year evolution of upper limb chorea in HD. Across four studies, 1025 participants collected smartwatch accelerometer data during their everyday life. We developed a machine learning model anchored to in-clinic upper limb chorea and trained cross-sectionally on disease-relevant features. The final model, selected using nested cross-validation, allowed estimating the longitudinal evolution of chorea. The resulting digital passive monitoring chorea score shows a progression of 0.13 points per year (95% CI: [0.08, 0.17]), and a robust sensitivity to change across studies, avoiding biases related to in-clinic measurements. High-resolution accelerometer data collected passively during daily life can improve the measurement of chorea in HD. Clinical Trial Registry Name: ClinicalTrials.gov; Study IDs: NCT03761849 registered 2018-11-30 and NCT03664804 registered 2018-09-07.

## Introduction

Huntington’s disease (HD) is a rare autosomal dominant neurodegenerative disease affecting the ability to function through the deterioration of motor, cognitive and behavioural functions^1^.

Chorea is a prominent symptom of HD, reported in more than 90% of patients^2,3^. It is characterized by hyperkinetic involuntary movements, often affecting patients’ extremities and facial muscles in early disease stages, before spreading to the limbs and trunk^4^. Chorea manifests throughout the day, and progressively interferes with patients’ daily activities^5^. However, its impact extends beyond the motor domain: in addition to its impact on the ability to function independently, visible motor dysfunction leads to stigma and social isolation^6,7^. Consequently, 17% of people with HD in a 2500-participant survey reported chorea to be the most impactful HD symptom^8^.

Effectively managing chorea requires reliable measurements to capture its severity and progression. Chorea is typically assessed during the Total Motor Score (TMS) examination, a component of the Unified Huntington’s Disease Rating Scale (UHDRS)^9^. However, this measure has several limitations: it does not capture day-to-day variability, relies on subjective assessments – leading to high inter-rater variability^10^ and placebo effects in clinical trials^11–13^ – and does not track subtle or infrequent chorea manifestations^11,14^. Developing more objective, frequent and sensitive measures of chorea is therefore key to better managing it^15^.

Digital solutions are emerging to address these measurement limitations^16–20^. A digital measure of chorea using a grip instrument has been deployed in clinical trials and observational studies^11,15,21,22^, showing increased objectivity and robustness to placebo effects^12^. However, being non-remote, it does not provide information about the evolution of chorea between clinical visits.

Smartphone or wearable-based measurements can complement in-clinic assessments by providing frequent measures taken in the participant’s natural environments. Clinical evaluations are essential especially in the diagnostic context. However, they are limited by their episodic nature and the inherent subjectivity of clinician-rated scales. Furthermore, clinic-based scores may not fully reflect a patient’s average state, and instead reflect specific measurement circumstances, as symptoms like chorea can sometimes be transiently exacerbated by the situational anxiety often associated with medical visits^23^. Task-based measures have previously been collected using smartphones^24,25^, and recently allowed the development of a digital motor score (the HDDMS), combining data from multiple motor functions to assess the overall progression of motor manifestations in HD^19^. Additionally, accelerometer data collected using wearables, such as smartwatches, could provide continuous insight into HD symptoms during daily life, in a low-burden way^20^.

Several studies have investigated the use of accelerometer data to measure clinical signs of HD. In-clinic, such digital scores showed promising results in distinguishing people with HD versus gene-negative controls^26–28^, or predicting clinical scores^28–30^. One study reports a chorea score, derived from data collected during task-based assessments, showing that chorea can be estimated from sensor data^31^. Some studies additionally analysed data collected during participants’ daily lives and showed that gait parameters distinguished between people with HD and gene-negative participants^32^, and were associated with HD severity^33^. Finally, one study followed patients over time and derived a truncal chorea index reflecting the amplitude of jerky movements from adhesive accelerometer devices^34^. However, they found no association between the digital chorea index and cUHDRS, and detected no statistically significant evolution of this chorea index over a 12-month period in a sample of 15 participants with HD. Further innovation is therefore needed to develop a digital score capable of sensitively tracking the progression of chorea.

In this study, we developed a Digital Passive-monitoring Chorea Score (DPCS), using a cohort of over 1000 participants who collected smartwatch accelerometer data during their everyday life for up to two years. The score was obtained by training a machine learning model to cross-sectionally estimate upper limb chorea scores as assessed in the clinic during the TMS examination. We applied the score to unseen longitudinal data from the placebo arm of the GENERATION HD1 tominersen clinical trial, and evaluated its capacity to estimate the longitudinal progression of upper limb chorea.

## Results

### Data collection and passive monitoring adherence

Participants from four studies (HD Natural History Study (NHS), Digital-HD^35^, GENERATION HD1, a double-blind phase III study designed to test the efficacy and safety of tominersen in HD patients^36^: original protocol (GENERATION HD1 OP), and final protocol (GENERATION HD1), see Supplementary Tables 1-2 for inclusion criteria and demographic characteristics of the participants) were equipped with smartwatches and collected wrist accelerometer data during their daily lives. 1025 participants collected on average 7.85 h of passive monitoring data per day, 5.75 days a week, for up to two years. At baseline, 78% of the participants of Digital-HD, 91% of NHS, 94% of GENERATION HD1 OP and 88% of GENERATION HD1 had sufficient passive monitoring data to be included in the analysis (Table 1). After 4 months, 85% of participants who collected data at baseline still collected sufficient data (73% of Digital-HD, 96% of NHS, 67% of GENERATION HD1 OP and 87% of GENERATION HD1). This number dropped to 72% for NHS and 55% for GENERATION HD1 after 12 months, and finally 15% of the participants of GENERATION HD1 (106 participants) still provided data 2 years after baseline (Table 1 and Supplementary Fig. 1). Note that passive monitoring in these studies was accompanied by daily active tasks administered by smartphone, and most participants were enrolled in an intensive trial of an intrathecal therapeutic, which may have impacted their adherence.

**Table 1:** Passive monitoring adherence

			Months from baseline												
		Enrolled	0	2	4	6	8	10	12	14	16	18	20	22	24
**Digital-HD**	***N***	120	93	73	68										
	**% enrolled**		78	61	57										
	**% baseline**		100	78	73										
**GENERATION HD1 OP**	***N***	107	101	89	68										
	**% enrolled**		94	83	64										
	**% baseline**		100	88	67										
**GENERATION HD1**	***N***	786	693	648	606	568	477	456	384	300	251	214	189	140	106
	**% enrolled**		88	82	77	72	60	58	49	38	32	27	24	18	13
	**% baseline**		100	94	87	82	69	66	55	43	36	31	27	20	15
**NHS**	***N***	78	71	69	68	59	52	55	51						
	**% enrolled**		91	88	87	76	67	71	65						
	**% baseline**		100	97	96	83	73	77	72						
**Total**	***N***	1091	958	879	810	627	529	511	435	300	251	214	189	140	106
	**% enrolled**		88	80	74	57	48	47	40	27	23	20	17	13	10
	**% baseline**		100	92	85	65	55	53	45	31	26	22	20	15	11

*N* Number of participants enrolled in the studies and equipped with a Ticwatch E, or collecting sufficient passive monitoring data to be included in the analysis for different months following baseline assessment. % enrolled and % baseline: percentage of N with respect to the number of participants enrolled in the study, or collecting sufficient data at baseline (month 0).

In addition to passive monitoring data collection, all participants underwent in-clinic assessments approximately every 3 months. Trained clinicians provided ratings of participants’ upper limb chorea on a scale from 0 (absent) to 4 (marked and prolonged) as part of the UHDRS assessment. These ratings, averaged across the left and right limbs, were used as anchors in the machine learning training.

### Linear regression with 22 features enables robust chorea estimation

**Fig. 1:**
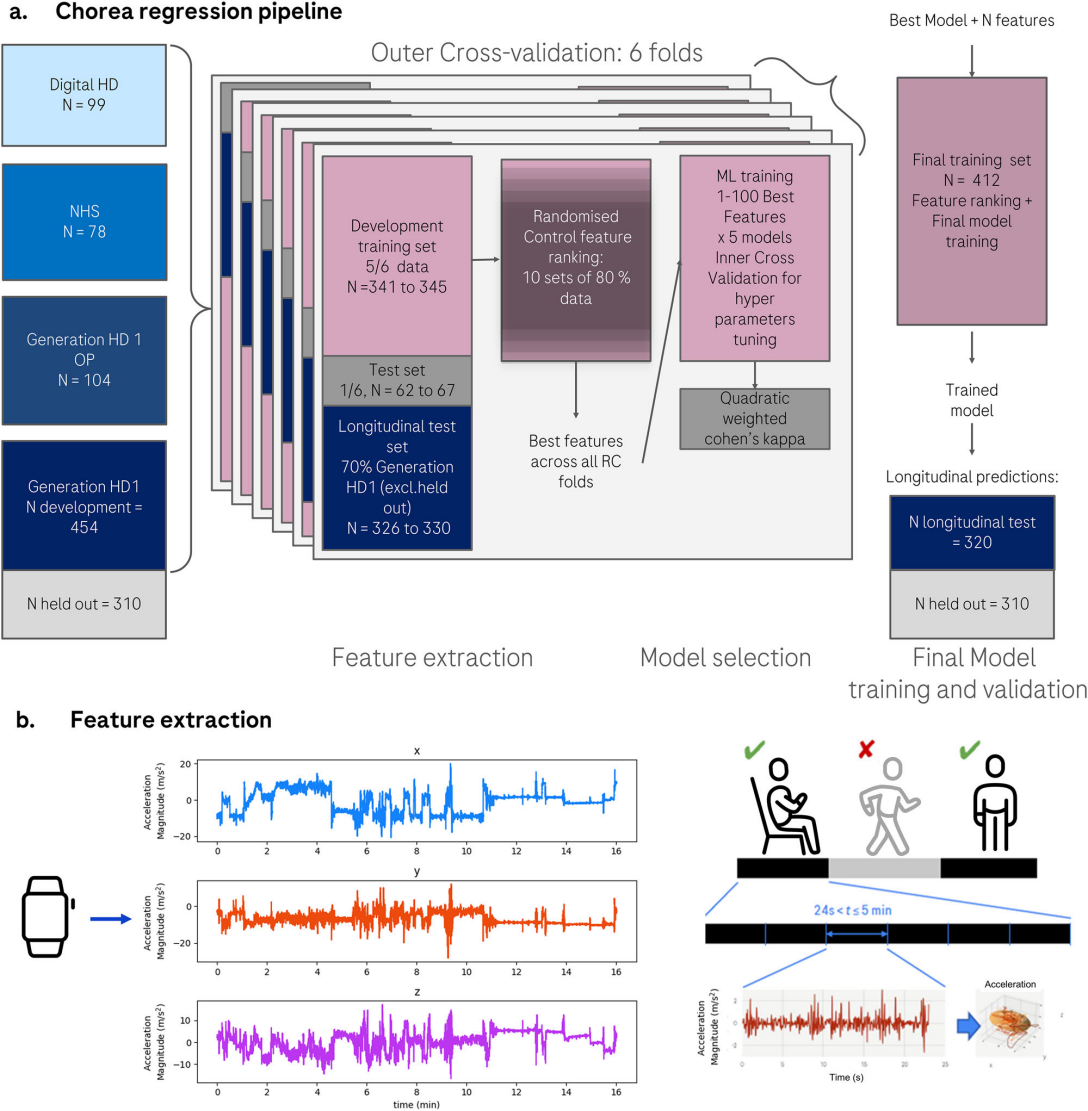
Estimating in-clinic Chorea from Passive Monitoring signal: pipeline and feature extraction. **a** Datasets and machine learning pipeline: features from 4 studies were extracted. After setting aside 310 participants for final testing, the data was split into training and test sets, using a 6-fold cross-validation procedure. This allowed the evaluation of feature selection methods and the selection of the best-performing model on unseen data. The final model was trained using data from both training and testing sets, excluding held-out and longitudinal test participants, then tested on the excluded participants. **b** Feature extraction: raw tri-axial accelerometer (left) was used to compute vector magnitude (right). Features were computed using non-gait periods identified using a human activity recognition algorithm and aggregated over two-week periods.

**Fig. 2:**
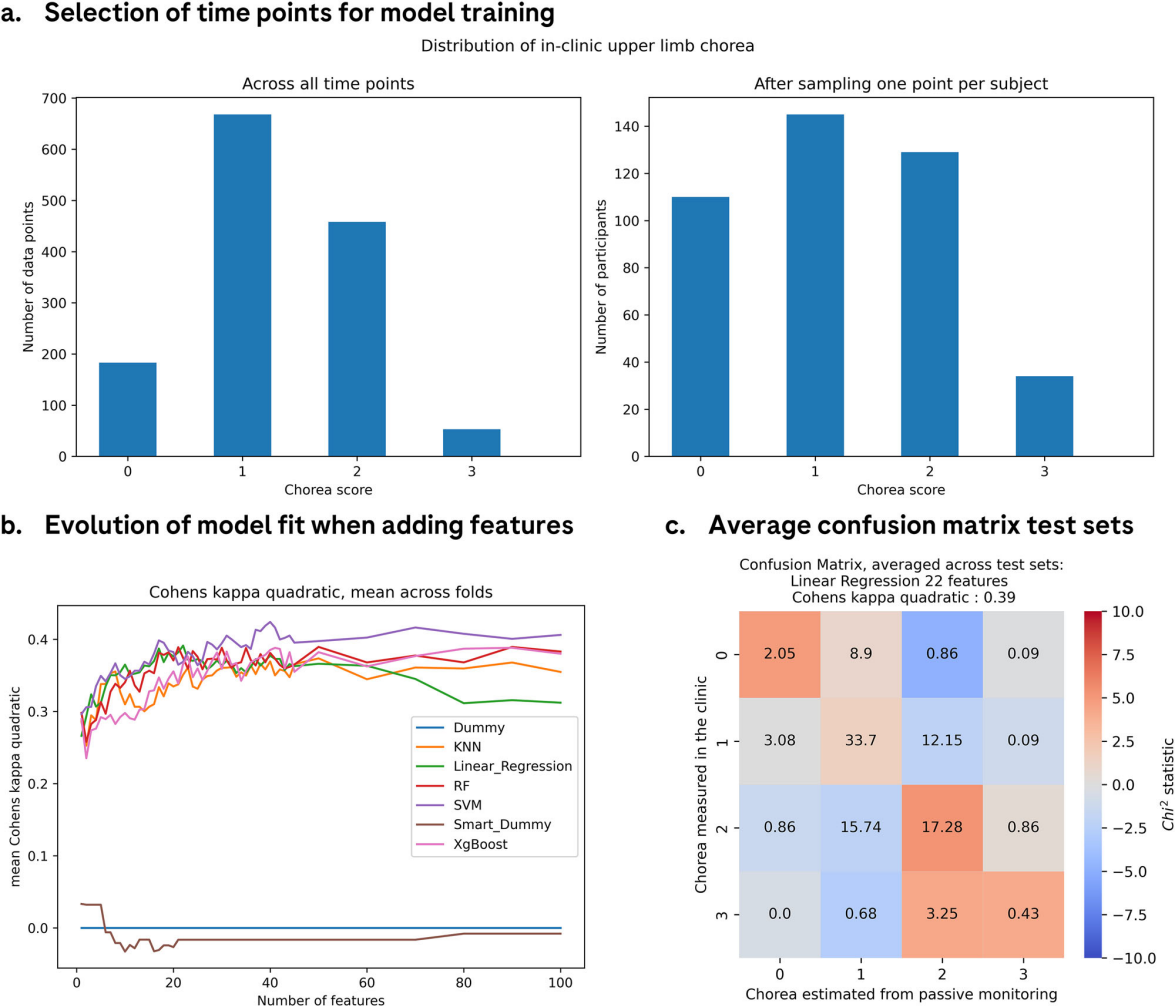
Machine learning: selection of training data and best-performing model. **a** Distribution of in-clinic chorea scores in training sets across all 4 datasets (left) and after selecting one time point per participant (right). Time points with underrepresented chorea scores were selected more frequently to limit biasing the models towards over-represented chorea classes. **b** Evolution of different model performance as features are iteratively added. Model performance is defined as the mean quadratic weighted Cohen’s kappa (penalizing predictions deviating from in-clinic scores by more than one point) across the 6 outer cross-validation test sets (i.e. 6 iterations of training and testing models on different datasets). The models tested were: linear regression; KNN: K-nearest neighbor; RF: Random Forrest; SVM: Support Vector Machine; XgBoost; and two dummy models, estimating the median of the data (Dummy), or the most likely class (Smart dummy). See Supplementary Fig. 6 for a representation including standard deviations across cross-validation folds. **c** Confusion matrix representing the predictions of the best type of model (Linear regression with 22 features), averaged across all 6 outer cross-validation test sets. The numbers represent the average number of participants in each category across the 6 folds (DPCS on the x axis vs in-clinic chorea on the y axis). The colors represent the Chi^2^ statistics: (Observed - Expected)^2^ / Expected, where Observed is the number of participants in each category, and Expected is the number of participants in a category one would obtain if the in-clinic and passive monitoring chorea scores were drawn from independent distributions. The Chi^2^ statistic was multiplied by the sign of the (Observed - Expected) difference, so that observations more (resp. less) frequent than expected under the independence hypothesis are represented in red (resp. blue).

To approximate in-clinic measures of upper limb chorea from passive monitoring, machine learning models were trained using uncorrelated features selected based on disease-related criteria (see Methods). The models were trained using data from different studies, leaving 310 participants out for final validation. A nested cross-validation was used to evaluate the performance of different models (Fig. 1a). A linear regression with the 22 highest-quality features (combination of congruent validity, correlation with in-clinic chorea, and test-retest reliability^19^, see Methods and Supplementary Fig. 2–5) was identified as having the best combination of accuracy, parsimony, and robustness to overfitting. It achieved an average quadratic weighted Cohen’s kappa of 0.39 across the 6 test sets (Fig. 2b, Supplementary Fig. 6). A more complex SVM regression model including 40 features achieved a higher Cohen’s kappa (0.42) but showed signs of overfitting (Supplementary Fig. 7), while linear regression models exhibited minimal overfitting. Given the overall comparable performance across models (Fig. 2b), the combination of accuracy, simplicity, and robustness made linear regression the best trade-off for a translational biomarker.

The 22 selected features belong to four categories, characterizing distinct dimensions of the movement signal. Complexity-based features quantified the involuntary unpredictability and chaotic nature of choreic jerks. Magnitude features captured the total intensity and spatial extent of the hyperkinetic displacement. Stability features measured the frequent signal fluctuations and loss of postural control during activity. Frequency-based features distinguished the specific signatures of chorea from the lower-frequency components of voluntary movement. While these features were not validated against identified chorea episodes in the current study, their data-driven selection should allow for a combination that captures the multifaceted, stochastic nature of chorea across various disease stages. The complete feature list is provided in Supplementary Table 3.

### DPCS approximates in-clinic upper limb chorea

**Fig. 3:**
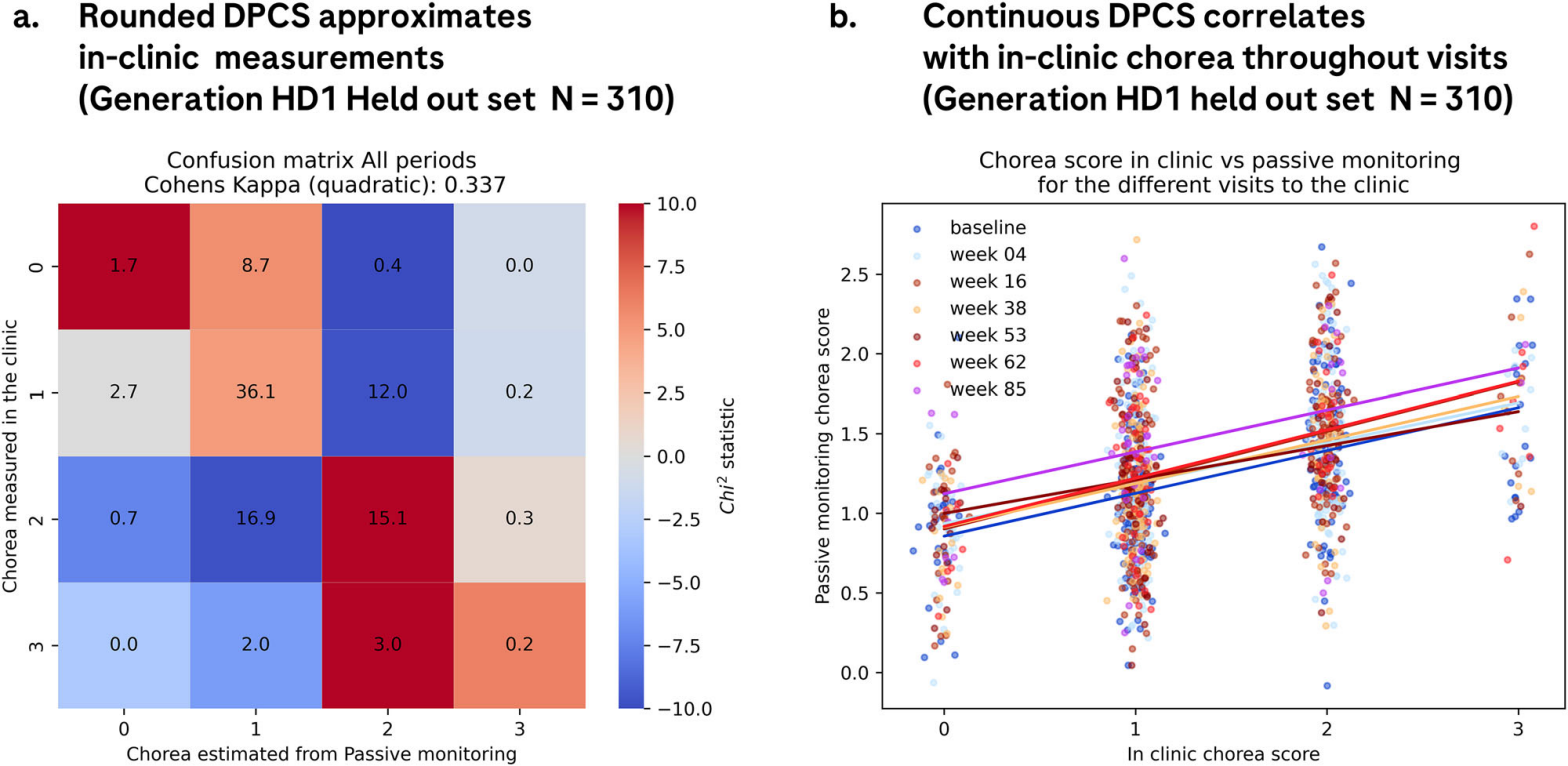
DPCS approximates in-clinic chorea measurement. **a** Confusion matrix representing the estimations of the final model (linear regression with 22 features), on held-out data. The numbers represent the percentage of participants in each category (DPCS on the x axis vs in-clinic chorea on the y axis). The colors represent the Chi^2^ statistics multiplied by the sign of the (Observed - Expected) difference. This statistic measures the expected agreement between in-clinic and passive monitoring scores, assuming independence, accounting for distribution imbalance. Observations more (resp. less) frequent than expected under the independence hypothesis are represented in red (resp. blue), showing that the model was within one point more often than expected under independence, and deviated by two or more points less frequently. **b** Cross-sectional relation between in-clinic chorea and DPCS for the different visits to the clinic. Each point represents a measurement for one participant. The lines represent a linear regression between the two scores. The colors represent the different visits.

After training the final model, we evaluated its cross-sectional performance for the 310 held-out participants. As in-clinic assessments provide a discrete score, the continuous model predictions were rounded to the nearest integer for this validation. A confusion matrix was constructed to compare the chorea score estimated from the model with the score measured in the clinic (Fig. 3a). The model correctly estimated the in-clinic chorea score for 53.1% of the observations, was off by 1 point for 43.7% of the observations, and only rarely deviated by more than 1 point (3.2%), approximating levels of agreement expected when different clinicians evaluate chorea in the same patients^10^. The final model achieved a Cohen’s kappa of 0.34, substantially higher than chance level (0 by definition; average across 1000 models trained using random permutations of the labels: 0.018, 95% CI: [0.009, 0.027], Supplementary Fig. 8).

The passive monitoring model produces a continuous score, providing more granular measurement than the discrete in-clinic assessments. After validating the accuracy of the rounded DPCS, we evaluated the continuous score. The model predictions significantly differed from chance predictions (Supplementary Fig. 8b, Mann-Whitney U test *p* < 0.001), except for predictions of an in-clinic chorea score of 1 (*p* = 0.09). While the DPCS score was closer to the correct in-clinic scores than chance, it overestimated chorea scores of 0 and 1, and underestimated scores of 2 and 3. This observation is characteristic of regression-to-the-mean errors^37^, and linked to the estimation of discrete, bounded variables^38^, where mathematical limits at 0 and 3 force all prediction errors to point toward the center of the scale.

As participants attended multiple visits to the clinic, we assessed the correlation between in-clinic and continuous DPCS across different visits (Fig. 3b). The average correlation was 0.42, consistent across all time points (Spearman correlations, *p* = 0.01 for week 85, *p* < 0.001 for all other visits; see Supplementary Table 4), showing that the DPCS consistently estimates in-clinic chorea throughout the trial.

### DPCS tracks chorea progression over time and across disease stages

After establishing the cross-sectional validity of the DPCS, we assessed its ability to measure the progression of chorea longitudinally and across different disease stages.

**Fig. 4:**
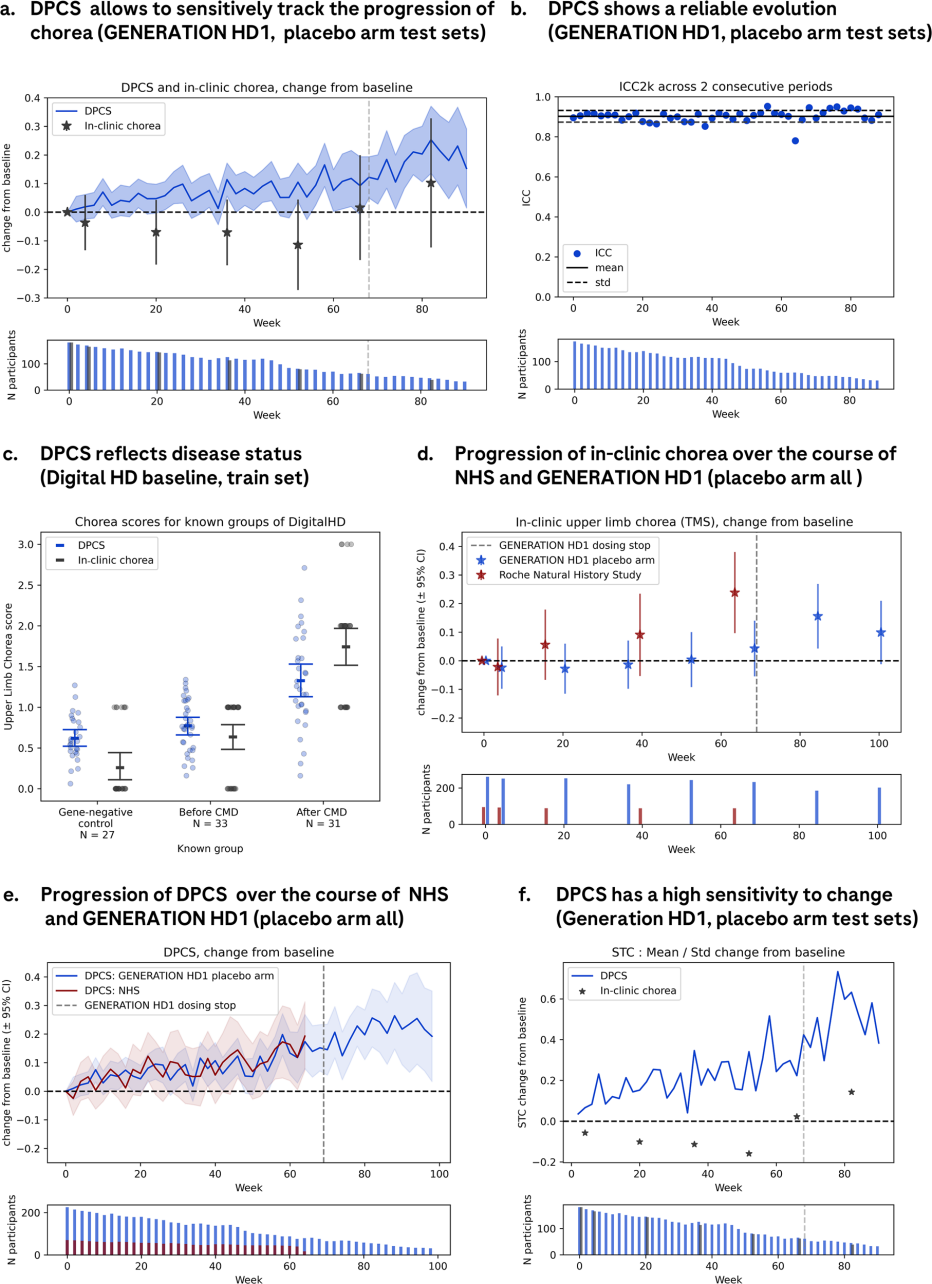
DPCS progresses over time in a disease-related manner, avoiding biases linked to visits to the clinic. **a** Evolution of DPCS (blue) and in-clinic chorea (grey) over the course of 98 weeks of the GENERATION HD1 clinical trial, for participants in the placebo arm who were not included in model training (*N* = 181). Error bars represent 95% confidence intervals. The bottom plot represents the number of participants who collected passive monitoring data at each time point. **b** Intra-class correlation coefficient (ICC(2,k)) computed for each consecutive fortnight, using all participants having passive monitoring data during both fortnights. Each dot represents one time point; the lines represent the average (plain line) and standard deviation (dashed line) across all 2-week epochs. The bottom plot represents the number of participants collecting data at each time point. **c** DPCS (blue) and in-clinic chorea (grey) at baseline for the different disease groups of the Digital-HD observational study (training set). CMD: Clinical Motor Diagnosis. Error bars represent 95% confidence intervals. **d** Progression of in-clinic chorea over the course of NHS and GENERATION HD1 (placebo arm). For each planned visit to the clinic, the upper limb chorea score (rounded average of the left and right arm scores) measured in the clinic is averaged across participants. The stars represent the average and error bars represent 95% confidence intervals. The bottom plot represents the number of participants for whom in-clinic assessments were recorded and that are included in the top plot. All participants in the placebo arm of GENERATION HD1 were included (*N* = 264), as well as all participants in NHS (*N* = 95), regardless of whether they collected passive monitoring data. **e** Progression of DPCS over the course of NHS (red) and GENERATION HD1 placebo arm (blue). Error bars represent 95% confidence intervals. The bottom plot represents the number of participants for whom passive monitoring was recorded and that are included in the top plot. All participants who recorded passive monitoring data were included (including training participants) for the placebo arm of GENERATION HD1 (*N* = 225), and NHS (*N* = 70). **f** Sensitivity to change (mean / standard deviation across participants) of the DPCS (blue) and in-clinic chorea (grey) for the placebo arm of the GENERATION HD1 clinical trial (test participants, *N* = 181). **a, d**, **e**, **f** The grey dotted lines represent week 68, when dosing stopped in the GENERATION HD1 clinical trial.

We first confirmed that the score significantly increased with time, consistent with the expected progression of HD chorea in all but end-stage HD^5,39^. We evaluated chorea progression using data from all placebo-arm participantss in the GENERATION HD1 clinical trial who were not included in model training (longitudinal test and held-out sets, *N* = 181, Fig. 4a). To account for the substantial drop-out of participants throughout the course of the study, the analysis was replicated using a subset of participants who continuously collected data between baseline and week 50, confirming an observed longitudinal progression of chorea in this subset (Supplementary Fig. 9a).

A linear mixed-effects model, taking into account imbalanced missing data across participants, quantified the score’s progression over the study duration, estimating a yearly increase of 0.13 points for the DPCS (95% CI: [0.08, 0.17], *p* < 0.001; Supplementary Table 5). The longitudinal progression replicated for the 68 weeks of the Natural History Study (Supplementary Fig. 10, Supplementary Table 6, yearly progression 0.15, 95% CI: [0.07, 0.23], *p* < 0.001). A model including participants from both studies showed no significant effect of the study or interaction between study and time on the DPCS change (Supplementary Table 7, study *p* = 0.23, interaction *p* = 0.60).

As a substantial proportion of participants with HD used symptomatic treatments to manage chorea (Supplementary Table 8), we conducted an exploratory analysis controlling for such treatments. We found no significant difference in the results when including this control (Supplementary Table 9).

We then verified that the yearly progression was plausible, with no abrupt changes^39^. An ICC, computed for each pair of consecutive fortnights in the placebo participants of GENERATION HD1 (*N* = 181), showed that the chorea progression was consistent over time (Fig. 4b. average ICC across periods 0.899, CI: [0.889, 0.909]).

The Digital-HD study additionally allowed us to compare the DPCS for known disease groups (Fig. 4c). The model applied to the baseline visit of Digital-HD showed different DPCS values for different disease stages (gene-negative controls, gene-positive participants before clinical motor diagnosis (CMD), and gene-positive participants after CMD). An ANOVA confirmed between groups differences in the DPCS (F(2, 88) = 22.83, *p* < 0.001). Post-hoc tests showed significant differences between manifest participants and controls or gene-positive participants pre-CMD (Welch’s *t*-tests, t(56) = *−*5.92 and t(62) = *−*4.59, *p* < 0.001 for both comparisons), but no significant differences between controls and gene-positive participants pre-CMD (t(58) = *−*1.97, *p* = 0.053). The mean expected time to motor diagnosis in these pre-CMD participants was estimated to be 11.3 years, using the Langbehn formula^40^.

A retrospective staging of participants in the HD-ISS staging system^41^ showed a significant difference between the DPCS of participants classified as HD-ISS stage 2 and HD-ISS stage 3 for the placebo arm of GENERATION HD1 (Supplementary Fig. 11, Welch’s t-tests: t(60) = *−*2.67, *p* = 0.010).

### DPCS is robust to in-clinic biases

The DPCS showed consistent longitudinal change of around 0.13 points per year in both the placebo arm of GENERATION HD1 (0.13, 95% CI: [0.08, 0.17]) and NHS (0.15, 95% CI: [0.07, 0.23], Fig. 4a, e, Supplementary Fig. 10). The rate of change was mostly constant over time, though increasing in variability as the number of data points decreased. In contrast, in-clinic chorea exhibited non-linear progression, with no detectable changes in the early phases of GENERATION HD1, and some progression only after week 85 (Fig. 4d). Note that dosing was halted after most participants had completed the visit at week 68 of GENERATION HD1 (see Supplementary Methods). For NHS, the progression of in-clinic chorea appeared to be more linear, and not limited to later visits, suggesting that the lack of progression of chorea during early phases of the GENERATION HD1 clinical trial may be due to some placebo bias related to interventional studies. The comparable progression of the DPCS in observational versus interventional studies confirms its robustness to such biases that confounded in-clinic motor assessments (Fig. 4e, Supplementary Table 7).

A consequence of such in-clinic bias is a low sensitivity to change (measured as the ratio between mean and standard deviation of change from baseline) of in-clinic chorea in GENERATION HD1 (Fig. 4f). Being robust to such bias, the DPCS has a consistently higher sensitivity to change, reliable across GENERATION HD1 and NHS (Fig. 4f, Supplementary Fig. 10b). A replication of analyses including only participants collecting data at all time points between week 0 and 50 confirmed the comparable progression of the DPCS across studies, and consistently high sensitivity to change, despite the smaller sample size (Supplementary Fig. 9b–d).

## Discussion

Using data from over 1000 participants with the HD genetic expansion and controls, we derived a Digital Passive Monitoring Chorea Score (DPCS), estimating the upper limb chorea of people with HD. The DPCS provides an approximation of chorea as assessed in-clinic during the UHDRS motor examination, performing significantly better than chance across the severity spectrum. While further work is needed to accurately estimate chorea of the highest and lowest severity, the score appears effective as a measure of change in participants with overt motor symptoms of HD. Our findings suggest that the DPCS sensitively measures the progression of chorea, estimating a yearly increase of around 0.13 points (95% CI: [0.08, 0.17]). For comparison, the progression of upper limb chorea evaluated during the TMS in this population is around 0.10 points per year. The DPCS was robust to placebo effects, progressing at the same rate for both observational and interventional studies, and consistently exhibited a higher sensitivity to change than traditionally used in-clinic chorea measurements. Because its development was anchored to UHDRS chorea scores, DPCS values are intuitive and easy to understand. This study highlights the potential of passive monitoring for assessing the clinical signs of HD.

Digital biomarkers derived from passive monitoring could serve useful roles in the development and use of potential treatments in HD: monitoring the emergence and severity of chorea; assessing the response to chorea medications; and potentially serving as trial endpoints, possibly allowing for earlier, shorter, and smaller trials^21,42,43^. The development of passive-monitoring biomarkers for HD is still in its early stages, and its potential needs to be affirmed through further study. Here, we propose such a demonstration and test the feasibility of monitoring chorea through accelerometer data.

We show that passive monitoring can mitigate some of the limitations of in-clinic assessment. First, the DPCS effectively alleviates the strong inter-rater variability observed in in-clinic measures of chorea^10^. Training a model using data collected from several hundred participants across 18 countries reduces biases introduced by individual raters or participants, resulting in a more objective tool. The DPCS shows a good agreement with the in-clinic measure, diverging by at most one point for the vast majority of observations (96.8%), replicating the expected inter-rater agreement for motor signs in neurodegenerative diseases^10,44,45^.

Second, the DPCS is robust to in-clinic biases and effects associated with interventional studies^13,22^. As previously reported for other in-clinic measures in GENERATION HD1^13^, the in-clinic measure of upper limb chorea was susceptible to placebo effects. Unlike chorea measured in observational studies, it did not progress during the early phases of the trial and only returned to levels observed in the Roche Natural History Study after the trial was halted. The DPCS, on the other hand, did not show differences in progression between interventional and observational studies. With ongoing efforts to reduce placebo treatments in clinical trials of rare diseases^46^, and the increasing use of external comparator control arms for licensing decisions^47,48^, such effects should be cautiously considered. Digital measures could provide a better way of comparing the progression of HD during clinical trials and external control studies.

In addition to reducing measurement bias, the DPCS showed the potential of passive monitoring to sensitively track thelongitudinal evolution of chorea. Previous research using accelerometer data to measure the signs of HD successfully distinguished between gene-negative controls and participants with HD^32^, or correlated accelerometer features with clinical scores^33^, but had so far not captured significant longitudinal evolution^34^. In the present study, we derived the DPCS from cross-sectional data, based on chorea severity, and showed that the resulting score could longitudinally track chorea. Our measured progression of 0.13 (95% CI: [0.08, 0.17]) points per year on a four-point scale corresponds closely to the 1-point progression of total maximum chorea on a 28-point scale previously reported for people with early-stage HD^49^, which translates to 0.14 per year on a four-point scale. These results demonstrate the potential of passive monitoring to measure the evolution of the signs of HD.

This study is not without limitations: first, the DPCS was constructed as a data-driven model, based on unlabeled data, trained against in-clinic measurements. We could not verify that the accelerometer features specifically derive from choreic movements. In addition to chorea, other movement disorders can affect the upper limb function of HD participants. It is thus possible that the DPCS captures clinical signs that are highly correlated with the presentation of chorea in the clinic, rather than chorea per se. However, the creation of a large dataset with annotated chorea events – especially subtle ones – captured during typical everyday activities, which would be required to create a more confidently chorea-specific score, may not be practical.

The datasets used in this study, while large and diverse, are limited by their scope: the DPCS was developed using data from studies with specific inclusion criteria (Supplementary Tables 1 and 2), which may not represent the symptom distribution of the general HD population. Including participants with a wider range of disease severity in future studies could improve model generalizability and provide insights into chorea progression across different stages^50^. Expanding the cohort to include a higher proportion of participants at the severity extremes would also mitigate the observed regression towards the mean^37^ by reducing the structural bias introduced by our current sample’s concentration around scores 1 and 2^51^. Beyond these distribution effects, the current analysis is limited to wrist motion. The DPCS is therefore not intended to capture the earliest signs of chorea manifesting in digits, toes, or face^4^, and is currently of limited use for participants of HD-ISS stage 0 or 1. Instead, the tool is designed for monitoring upper limb chorea in research cohorts, either to specifically assess chorea, or to complement other measures of Huntington’s disease.

A notable limitation of the current study is the decrease in participant adherence over time. Previous analyses of these patterns (see^36^ supplementary appendix, and^52^), revealed that data collection frequency was influenced by both disease severity (cUHDRS) and the participants’ subjective perception of their health status (EQ-5D visual analogue scale^53^). While further research is required to fully characterize the causes of reduced adherence, a key factor was likely the high participant burden. In the present studies, passive monitoring was paired with extensive daily active motor tests, creating a demanding schedule. If deployed as a standalone measure or alongside a smaller battery of active tasks, as well as using a device that does not require daily charging, the participant burden should be substantially reduced, and we anticipate a concomitant improvement in adherence. Despite this data missingness, the DPCS remained capable of detecting significant longitudinal changes. Notably, the score exhibited high sensitivity to change even with a reduced amount of longitudinal data compared to traditional in-clinic measurements. These results suggest that the DPCS is robust to incomplete data, though future studies are needed to evaluate the DPCS performance in datasets without potentially biased data missingness.

Finally, several symptomatic treatments exist to lower chorea symptoms^54,55^. While the present data did not detect a significant impact of such treatments on the cohort progression of chorea, the underlying studies were not designed to study these effects. Various treatments and potential dose changes may affect the evolution of chorea for different participants, and their differential impact on DPCS progression would need to be considered. A follow-up study designed specifically to evaluate changes in DPCS with symptomatic treatments would allow to assess the specificity of DPCS in measuring chorea.

Although further studies are needed to validate the DPCS as a reliable objective proxy for chorea, its robustness to in-clinic biases, and its ability to distinguish disease stages and capture longitudinal progression of chorea across studies support its potential use for objectively measuring chorea progression in future studies. Robustness to in-clinic biases further suggests that, following additional validation, the DPCS might be considered for use in external comparator control arms in interventional trials.

In conclusion, with this proof-of-concept study, we show that passive monitoring allows the estimation of chorea during the everyday life of people with HD, in a way that is robust to in-clinic biases. Wearables offer promising and low-burden options to evaluate the progression of signs of HD and other neuromuscular diseases, and could allow a better understanding of the effects of potential disease modifying or symptomatic treatments.

## Methods

### Studies

Data from 1113 participants was collected across four studies (HD Natural History Study (NHS), Digital-HD^35^, GENERATION HD1, a double-blind phase III study designed to test the efficacy and safety of tominersen in HD patients^36^: original protocol (GENERATION HD1 OP), and final protocol (GENERATION HD1); see Supplementary Tables 1 and 2 for inclusion criteria and participants characteristics). Participants from all arms of GENERATION HD1 were used to develop and train machine learning models; participants from the placebo arm only were used to evaluate the longitudinal evolution of chorea.

### Passive monitoring collection

Passive monitoring data was collected as part of the Roche Remote Digital Monitoring Platform^24^, consisting of smartphone-based *active tasks* (i.e., tasks requiring active input by the user), and smartwatch-based *passive monitoring* (i.e., sensor placed on participants’ wrists, passively and continuously collecting data without active input by the user, during everyday life) and patient-reported outcomes.

Commercial smartwatches (Ticwatch E) were used to record tri-axial acceleration data from participants’ wrists at a sampling rate of 50 Hz. Participants were instructed to charge the devices overnight.

Twenty-two participants were equipped with a different device model and thus excluded from the current analysis (17 participants from NHS, 5 from GENERATION HD1). Additionally, 66 participants (23 from Digital-HD, 3 for GENERATION HD1 OP and 40 from GENERATION HD1) had insufficient clinical data (no visit recorded) or passive monitoring adherence (no day with at least 60 min of passive monitoring) to be included in the analysis.

Passive monitoring features were extracted for the different studies based on study duration and participants’ adherence (Supplementary Fig. 1): 32 weeks for GENERATION HD1 OP, 98 weeks for GENERATION HD1, 76 weeks for NHS, and 50 weeks for the Digital-HD study. The GENERATION HD1 study had a duration of 101 weeks, but the dosing was halted after most participants had completed the clinical visit scheduled on week 69, following a risk-benefit assessment conducted by an independent data monitoring committee.

### Clinical measurement of chorea

Trained raters provided a total of 6506 clinical evaluations of the participants during visits to the clinic using the UHDRS, rating chorea in 7 body regions on a scale from 0 (absent) to 4 (marked and prolonged). Assessments were taken at baseline for all participants, and subsequent assessments approximately every 3 months for NHS (5 visits) and GENERATION HD1 (original protocol: 4 visits, final protocol: 8 visits). Only the baseline assessment was analysed for Digital-HD due to difficulties in conducting regular follow-up clinical visits during the COVID-19 pandemic^35^. Subsequent visits occurred only after approximately 12 and 18 months for this study.

As the smartwatch collects accelerometer data from the wrist, the current analysis primarily reflects upper limb chorea and is not designed to capture the earliest or most subtle choreic manifestations, which may occur in the digits, toes, or face^4^. Consequently, to construct the in-clinic chorea score, the scores for the left and right upper limbs were averaged and rounded to the nearest integer. This aggregation was selected because participants wore the device on their wrist, and Huntington’s Disease (HD) chorea is not typically lateralized^56^. Across all collected data, only 30 instances had a chorea score of 4 (0.45% of the visits to the clinic). Owing to their sparsity, chorea scores of 4 could not be analysed separately and were therefore excluded from the analysis.

The in-clinic chorea score was used as a clinical anchor to train machine learning models. Despite the known inter-rater variability^10^, and potential biases, it represents the best available information regarding chorea severity in this dataset. While we do not expect machine learning models to perfectly fit this noisy score, they should provide a more intrinsically consistent measure.

### Machine learning pipeline overview

Accelerometer data collected during participants’ everyday life was used to train models estimating in-clinic chorea scores (Fig. 1a). Features summarizing different aspects of the signal over two-week periods were extracted from the passive monitoring data. A set of 100 features was selected using a maximum relevance, minimum redundancy ranking^57^ based on properties associated with chorea. One to 100 ranked features were then used to train a variety of machine learning models using selected time points (Fig. 3a). The models were tested using a 6-fold nested cross-validation procedure, and the type of model performing best on unseen data was selected (Fig. 3b). A final model was then trained using all data from the development set and applied to estimate the longitudinal evolution of chorea in held-out participants in GENERATION HD1.

### Feature extraction

The daily data coverage for each participant was computed, and only days with a minimum of 60 min of active wear-time were included in the analysis to ensure sufficient data density for feature calculation. This threshold was informed by the observation that data missingness primarily occurred as missed days or a total discontinuation of device use, rather than insufficient data collection within a single day^36^.

A wear algorithm was used to select the segments of data during which the watch was likely to be worn by the participant, and a human activity recognition algorithm^58^ was applied to detect non-gait segments (sitting, standing, or lying), which were used for this analysis (Fig. 1b). The data was split into continuous segments (gaps were defined as data missing for more than 1000 ms). To deal with the occasional non-constant sampling frequency observed in the devices used, the data was resampled to 35 Hz in order to have sufficient temporal resolution to detect sudden movements. The Euclidean Norm Minus One^59^ of the signal was computed as $\sqrt{{x}^{2}+{y}^{2}+{z}^{2}}-g$, where x, y, and z are the three axial acceleration signals in $m.{s}^{-2}$, and g the gravitational unit (g = 9.80655 $m.{s}^{-2}$). For each bout of activity, statistical features as well as Fast Fourier Transform and features previously reported in ref. ^24^ were extracted, then aggregated over days and, finally, over 14 days using a comprehensive set of statistics including mean, median, median absolute deviation, max, mode, standard deviation, skewness, kurtosis, and percentiles, resulting in approximately 11000 candidate features.

### Quality-based feature selection

To limit the risk of spurious associations, the most promising 100 out of the 11,000 candidate features were selected using the training data (Fig. 1a: randomized control feature ranking), based on properties associated with chorea in HD. First, feature normality was assessed using a Shapiro-Wilk test, and features were log-transformed if this improved their normality at baseline.

Second, features were filtered based on the congruence of their evolution over time and across known disease stages^19^. The progression of each feature over time was computed using NHS participants. The values of each feature for known disease groups (gene-negative controls, gene-positive participants before clinical motor diagnosis (CMD), and gene-positive participants after CMD) were computed using Digital-HD, and a slope was fitted representing the evolution of the feature across disease stages. Features with an incongruent progression (slopes of opposite sign for the two studies) were excluded (Supplementary Fig. 2, for more details about the congruence step, see ref. ^19^). Features containing outlier values (defined as values lying outside 10 times the interquartile interval), or fewer than 50 different values, were also removed.

Third, two quality measures were computed, quantifying the features’ association with in-clinic chorea (cross-cectional correlation (CSC), Supplementary Fig. 3a), and test-retest reliability (intraclass correlation coefficient (ICC), Supplementary Fig. 3b). The product of these two measures was used as a quality metric for each feature (see^19^ for details of the CSC and ICC measurement).

To ensure that the feature quality was independent from the participants used to evaluate it, the quality metric was computed for 10 random samples composed of 80% of the training participants (Supplementary Fig. 4). Features that had a signal-to-noise ratio (average quality metric divided by the standard deviation across the 10 random samples) below 2 were excluded.

Finally, the features were ranked using a minimum redundancy maximum relevance algorithm^57^, maximizing the quality metric averaged across the 10 random samples, and minimizing the correlation between features, to identify features with a high quality, while avoiding redundant information (see Supplementary Fig. 5 for correlations between top-ranking features). Up to 100 top-ranked features were used to train machine learning models. Features were ranked separately for each outer fold of the nested cross-validation procedure (Fig. 1a).

### Model selection: nested cross-validation

To evaluate different machine learning models while preserving a large amount of data for training a final model, we used nested cross-validation. First, 310 participants of GENERATION HD1 were set aside as a final held-out set. The data was then split in a stratified way into 6 non-overlapping outer cross-validation folds (Fig. 1a) used for evaluating the reliability and generalizability of the machine learning pipeline (see Stratification procedure).

The 6 folds were used to train different machine learning models (training sets), and to evaluate their performance on unseen data (test sets). An inner cross-validation loop was used to tune the hyperparameters of the different models. The performance of linear regression, random forest (RF), support vector machine (SVM), XgBoost, and K-nearest-neighbor (KNN) regression models was compared.

For each outer fold, features were ranked using the training data only. One time point per participant was then selected to ensure independent observations. To limit biasing the models towards over-represented chorea classes, time points with underrepresented chorea scores were selected more frequently (Fig. 2a, see Selection of time points for model training).

The different models were trained using 1 to 100 features, added sequentially based on their rank. The best type of model and number of features was identified as having the highest average quadratic weighted Cohen’s kappa across test sets (Fig. 2b), while controlling for overfitting (Supplementary Fig. 6-7).

### Stratification procedure

Participants were stratified in the different outer cross-validation folds, counterbalancing their in-clinic chorea at baseline, in-clinic chorea progression over time, and treatment arm for the GENERATION HD1 study. For each outer fold, 1/6th of the participants of NHS (N ≃ 16), Digital-HD (N ≃ 17), and GENERATION HD1 OP (N ≃ 17) formed the test set, while the remaining 5/6th participants formed the training set. As the GENERATION HD1 study had a larger number of participants, and to avoid over-representing one study in the model training procedure, a problem described by ref. ^60^, 70% of the participants (N ≃ 328) of this study were set aside as a longitudinal test set, while the remaining participants were split into 1/6th (N ≃ 17) for the test sets and 5/6th for the training sets. The number of participants in each fold slightly varied due to the stratification constraints.

To compute the stratification variables, a mixed-effects linear regression of in-clinic chorea on time including a random intercept and random slope for each participant was fitted. The random slope of each participant was used as a proxy for the progression of chorea for the participant. Participants were split into low, medium, and high progression using the tertiles of the distribution. Participants were stratified based on their chorea score at baseline, progression category, and treatment arm for the participants of the GENERATION HD1 clinical trial. The stratification was based on a random selection of participants producing a representative distribution of the stratification criteria. This random selection was repeated until the final training and longitudinal test sets had average chorea scores differing by at most 0.025 points at any time point.

### Selection of time points for model training

Participants took part in several visits to the clinic, receiving a chorea score at each visit. Models were trained using one time point per participant. One visit per participant was therefore selected for model training. To increase the number of data points for underrepresented chorea scores, and to train models able to distinguish between the different scores, a sampling procedure favoring under-represented scores was applied. Chorea scores were ranked based on their frequency, and scores with the lowest frequency were selected first, increasing their representation. First, as many of the least frequent chorea score (in-clinic chorea 3) as possible (with the limit of one per participant) were selected, then the procedure was repeated with the second least frequent score (in-clinic chorea 0) for the remaining participants, and finally, the last two scores (1 and 2) were alternately sampled until a time point for every participant had been selected.

### Final model training and validation

The winning model, selected during the cross-validation procedure, was trained using all participants of Digital-HD, NHS, and GENERATION HD1 OP, and 136 participants of GENERATION HD1, producing the final Digital Passive Monitoring Chorea Score (DPCS). The model was then applied to the remaining participants of GENERATION HD1 (longitudinal test set and held-out set), to evaluate its performance on unseen data, and used for longitudinal analyses of the placebo arm of GENERATION HD1 and NHS. To evaluate chance level performance, the same model was trained using random permutations of the in-clinic chorea labels, repeated 1000 times.

### Statistical analysis

Longitudinal model estimations were evaluated using mixed-effects linear regressions. The effect of time (expressed in years) on change-from-baseline (baseline score subtracted for each participant) was estimated using a mixed-effect model including participants as random intercepts and slopes, and age, CAG repeat length, sex, and score at baseline.

$${change}\,{from}\,{baseline}\, \sim \,{time}+{age}+{sex}+{CAG}\,{repeats}+{baseline}\,{score}\,+(1+{time|participant})$$

The coefficients and confidence intervals associated with time were used as estimates of yearly change from baseline.

### Ethics approval declaration

Written informed consent was obtained from all study participants prior to any study procedures. All data handling procedures were reviewed and approved by sponsors, funders and research ethics committees, and were compliant with all relevant data protection guidance and legislation. Data security management and sharing policies were described in the informed consent forms and agreed to by all participants. The studies were conducted in accordance with the Declaration of Helsinki.

Study protocols have been previously published for the NHS study (ClinicalTrials.gov Identifier: NCT03664804 registered 2018-09-07) and the GENERATION HD1 study (ClinicalTrials.gov Identifier: NCT03761849 registered 2018-11-30)^36^. These studies received ethics approval from central institutional review boards (GENERATION HD1: Supplementary Table 10, NHS: Supplementary Table 11). Digital-HD received ethics approval from the London-Central Research Ethics Committee (Reference: 18/LO/1986).

## Supplementary information

Supplementary Information

## Data Availability

Access requests for patient-level data from the placebo group of Generation-HD1 (BN40423) and the Natural History Study (BN40422) can be made via [Vivli](https://vivli.org/ourmember/roche). However, policy does not permit sharing of digital sensor data. Up-to-date details on Roche’s global policy on the sharing of clinical information and how to request access to related clinical study documents are available on the Roche [website](https://go.roche.com/data_sharing). Anonymized records for individual patients across more than one data source external to Roche cannot, and should not, be linked due to a potential increase in risk of patient reidentification.

## References

[1] Bates, G. P. et al. Huntington disease. *Nat. Rev. Dis. Prim.* **1**, 15005 (2015). 27188817 10.1038/nrdp.2015.5

[2] Anderson, K. E. Huntington’s disease. *Handb. Clin. Neurol* **100**, 15–24 (2011). 21496569 10.1016/B978-0-444-52014-2.00002-1

[3] Burgunder, J.-M. et al. An International Survey-based Algorithm for the Pharmacologic Treatment of Chorea in Huntington’s Disease. *PLoS Curr* **3**, RRN1260 (2011). 21975581 10.1371/currents.RRN1260 PMC3166256

[4] Roos, R. A. Huntington’s disease: a clinical review. *Orphanet J. Rare Dis* **5**, 40 (2010). 21171977 10.1186/1750-1172-5-40 PMC3022767

[5] Jankovic, J. & Roos, R. A. C. Chorea associated with Huntington’s disease: To treat or not to treat? *Mov. Disord.* **29**, 1414–1418 (2014). 25156927 10.1002/mds.25996

[6] Sherman, C. W., Iyer, R., Abler, V., Antonelli, A. & Carlozzi, N. E. Perceptions of the impact of chorea on health-related quality of life in Huntington disease (HD): A qualitative analysis of individuals across the HD spectrum, family members, and clinicians. *Neuropsychol. Rehabilitation* **30**, 1150–1168 (2020). 10.1080/09602011.2018.1564675 PMC7685000 30849283

[7] Thorley, E. M. et al. Understanding How Chorea Affects Health-Related Quality of Life in Huntington Disease: An Online Survey of Patients and Caregivers in the United States. *Patient* **11**, 547–559 (2018). 29750428 10.1007/s40271-018-0312-x PMC6132452

[8] Simpson, J. A., Lovecky, D., Kogan, J., Vetter, L. A. & Yohrling, G. J. Survey of the Huntington’s Disease Patient and Caregiver Community Reveals Most Impactful Symptoms and Treatment Needs. *J. Huntingt.’s Dis.* **5**, 395–403 (2016). 10.3233/JHD-160228 27983566

[9] Huntington Study Group Unified Huntington’s disease rating scale: Reliability and consistency. *Mov. Disord.* **11**, 136–142 (1996). 8684382 10.1002/mds.870110204

[10] Winder, J. Y., Roos, R. A. C., Burgunder, J., Marinus, J. & Reilmann, R. Interrater Reliability of the Unified Huntington’s Disease Rating Scale-Total Motor Score Certification. *Mov. Disord. Clin. Pr.* **5**, 290–295 (2018). 10.1002/mdc3.12618 PMC6174470 30363437

[11] Reilmann, R. et al. A randomized, placebo-controlled trial of AFQ056 for the treatment of chorea in Huntington’s disease. *Mov. Disord.* **30**, 427–431 (2015). 25689146 10.1002/mds.26174

[12] Reilmann, R. & Schubert, R. Motor outcome measures in Huntington disease clinical trials. *Handb. Clin. Neurol* **144**, 209–225 (2017). 28947119 10.1016/B978-0-12-801893-4.00018-3

[13] Boareto, M. et al. Modeling Disease Progression and Placebo Response in Huntington Disease. *Neurology* **104**, e213646 (2025). 40315394 10.1212/WNL.0000000000213646 PMC12057022

[14] Paulsen, J. S. et al. Challenges assessing clinical endpoints in early Huntington disease. *Mov. Disord.* **25**, 2595–2603 (2010). 20623772 10.1002/mds.23337 PMC2978744

[15] Reilmann, R., Bohlen, S., Kirsten, F., Ringelstein, E. B. & Lange, H. W. Assessment of involuntary choreatic movements in Huntington’s disease—Toward objective and quantitative measures. *Mov. Disord.* **26**, 2267–2273 (2011). 21661053 10.1002/mds.23816

[16] Dorsey, E. R., Papapetropoulos, S., Xiong, M. & Kieburtz, K. The First Frontier: Digital Biomarkers for Neurodegenerative Disorders. *Digit. Biomark.* **1**, 6–13 (2017). 32095743 10.1159/000477383 PMC7015357

[17] Adams, J. L., Waddell, E. M., Chunga, N. & Quinn, L. Biomarkers for Huntington’s Disease, Improving Clinical Outcomes. *Contemp. Clin. Neurosci*. 433–457 10.1007/978-3-031-32815-2_18 (2023).

[18] Tortelli, R., Rodrigues, F. B. & Wild, E. J. The use of wearable/portable digital sensors in Huntington’s disease: A systematic review. *Park. Relat. Disord.* **83**, 93–104 (2021). 10.1016/j.parkreldis.2021.01.006 PMC7957324 33493786

[19] Giboin, L.-S. et al. A digital motor score for sensitive detection of progression in Huntington’s disease. *Brain* **148**, 3228–3238 (2025). 10.1093/brain/awaf127 PMC12404731 40215265

[20] Quinn, L., Roché, M. W., Dorn, J. & Adams, J. L. The Digital Frontier in Huntington’s Disease: Opportunities for Clinical Trials. *Mov. Disord*. 10.1002/mds.30277 (2025). 10.1002/mds.30277 40658057

[21] Tabrizi, S. J. et al. Potential endpoints for clinical trials in premanifest and early Huntington’s disease in the TRACK-HD study: analysis of 24 month observational data. *Lancet Neurol* **11**, 42–53 (2012). 22137354 10.1016/S1474-4422(11)70263-0

[22] Reilmann, R. et al. Safety and efficacy of pridopidine in patients with Huntington’s disease (PRIDE-HD): a phase 2, randomised, placebo-controlled, multicentre, dose-ranging study. *Lancet Neurol* **18**, 165–176 (2019). 30563778 10.1016/S1474-4422(18)30391-0

[23] Wild, E. J. & Tabrizi, S. J. Premanifest and Early Huntington’s Disease’ in Huntington’s Disease. *Oxford**Univ. Press*10.1093/med/9780199929146.003.0005 (2014).

[24] Lipsmeier, F. et al. A Remote Digital Monitoring Platform to Assess Cognitive and Motor Symptoms in Huntington Disease: Cross-sectional Validation Study. *J. Med. Internet Res.* **24**, e32997 (2022). 35763342 10.2196/32997 PMC9277525

[25] Waddell, E. M. et al. GEORGE®: A Pilot Study of a Smartphone Application for Huntington’s Disease. *J. Huntingt.’s Dis.* **10**, 293–301 (2021). 10.3233/JHD-200452 33814455

[26] Acosta-Escalante, F. D., Beltran-Naturi, E., Boll, M. C., Hernandez-Nolasco, J. A. & Garcia, P. P. Meta-Classifiers in Huntington’s Disease Patients Classification, Using iPhone’s Movement Sensors Placed at the Ankles. *IEEE Access* **6**, 30942–30957 (2018).

[27] Dalton, A. et al. Analysis of gait and balance through a single triaxial accelerometer in presymptomatic and symptomatic Huntington’s disease. *Gait Posture* **37**, 49–54 (2013). 22819009 10.1016/j.gaitpost.2012.05.028

[28] Scheid, B. H. et al. Predicting Severity of Huntington’s Disease With Wearable Sensors. *Front. Digit. Health* **4**, 874208 (2022). 35445206 10.3389/fdgth.2022.874208 PMC9013843

[29] Bennasar, M. et al. Automated Assessment of Movement Impairment in Huntington’s Disease. *IEEE Trans. Neural Syst. Rehabilitation Eng* **26**, 2062–2069 (2018). 10.1109/TNSRE.2018.2868170 PMC6196596 30334742

[30] Bennasar, M. et al. Huntington’s Disease Assessment Using Tri Axis Accelerometers. *Procedia Comput. Sci.* **96**, 1193–1201 (2016).

[31] Gordon, M. F. et al. Quantification of Motor Function in Huntington Disease Patients Using Wearable Sensor Devices. *Digit. Biomark.* **3**, 103–115 (2019). 32095771 10.1159/000502136 PMC7011722

[32] Andrzejewski, K. L. et al. Wearable Sensors in Huntington Disease: A Pilot Study. *J. Huntingt.’s Dis.* **5**, 199–206 (2016). 10.3233/JHD-160197 27341134

[33] Keren, K. et al. Quantification of Daily-Living Gait Quantity and Quality Using a Wrist-Worn Accelerometer in Huntington’s Disease. *Front. Neurol.* **12**, 719442 (2021). 34777196 10.3389/fneur.2021.719442 PMC8579964

[34] Dinesh, K. et al. A Longitudinal Wearable Sensor Study in Huntington’s Disease. *J. Huntingt.’s Dis.* **9**, 69–81 (2020). 10.3233/JHD-190375 31868675

[35] Kinsella, F. C. et al. F029 Digital-HD: smartphone and smartwatch-based testing to assess the motor, cognitive, and behavioural symptoms of Huntington’s disease. *J. Neurol. Neurosurg. Psychiatry* **95**, A75–A76 (2024).

[36] McColgan, P. et al. Tominersen in Adults with Manifest Huntington’s Disease. *N. Engl. J. Med.* **389**, 2203–2205 (2023). 38055260 10.1056/NEJMc2300400

[37] Bland, J. M. & Altman, D. G. Statistic Notes: Regression towards the mean. *BMJ* **308**, 1499 (1994). 8019287 10.1136/bmj.308.6942.1499 PMC2540330

[38] Tobin, J. Estimation of Relationships for Limited Dependent Variables. *Econometrica* **26**, 24 (1958).

[39] Dorsey, E. R. et al. Natural History of Huntington Disease. *JAMA Neurol* **70**, 1520–1530 (2013). 24126537 10.1001/jamaneurol.2013.4408

[40] Langbehn, D. R., Hayden, M. R., Paulsen, J. S. & and the PREDICT-HD investigators of the Huntington study group CAG-repeat length and the age of onset in Huntington disease (HD): A review and validation study of statistical approaches. *Am. J. Med. Genet. Part B: Neuropsychiatr. Genet.* **153B**, 397–408 (2010). 10.1002/ajmg.b.30992 PMC3048807 19548255

[41] Tabrizi, S. J. et al. A biological classification of Huntington’s disease: the Integrated Staging System. *Lancet Neurol* **21**, 632–644 (2022). 35716693 10.1016/S1474-4422(22)00120-X

[42] Ross, C. A. et al. Huntington disease: natural history, biomarkers and prospects for therapeutics. *Nat. Rev. Neurol.* **10**, 204–216 (2014). 24614516 10.1038/nrneurol.2014.24

[43] Weir, D. W., Sturrock, A. & Leavitt, B. R. Development of biomarkers for Huntington’s disease. *Lancet Neurol* **10**, 573–590 (2011). 21601164 10.1016/S1474-4422(11)70070-9

[44] Morinan, G. et al. Computer vision quantification of whole-body Parkinsonian bradykinesia using a large multi-site population. *npj Park.’s Dis* **9**, 10 (2023). 10.1038/s41531-023-00454-8 PMC9883391 36707523

[45] Martinez-Manzanera, O. et al. A Method for Automatic, Objective and Continuous Scoring of Bradykinesia. *2015 IEEE 12th Int. Conf. Wearable Implant. Body Sens. Netw. (BSN)* 1–5 10.1109/bsn.2015.7299358 (2015).

[46] Chiodo, G. T., Tolle, S. W. & Bevan, L. Placebo-controlled trials: good science or medical neglect? *West. J. Med* **172**, 271–273 (2000). 10778385 10.1136/ewjm.172.4.271 PMC1070847

[47] Jahanshahi, M. et al. The Use of External Controls in FDA Regulatory Decision Making. *Ther. Innov. Regul. Sci.* **55**, 1019–1035 (2021). 34014439 10.1007/s43441-021-00302-y PMC8332598

[48] Thorlund, K., Dron, L., Park, J. J. H. & Mills, E. J. Synthetic and External Controls in Clinical Trials – A Primer for Researchers. *Clin. Epidemiology* **12**, 457–467 (2020). 10.2147/CLEP.S242097 PMC7218288 32440224

[49] Ravina, B. et al. The relationship between CAG repeat length and clinical progression in Huntington’s disease. *Mov. Disord.* **23**, 1223–1227 (2008). 18512767 10.1002/mds.21988

[50] Furr-Stimming, E. et al. Chorea Severity Change Over Time in Huntington Disease and by Huntington Disease Stage [abstract]. *Mov Disord.* 37, https://www.mdsabstracts.org/abstract/chorea-severity-change-over-time-in-huntington-disease-and-by-huntington-disease-stage/ (2022).

[51] He, H. & Garcia, E. A. Learning from Imbalanced Data. *IEEE Trans. Knowl. Data Eng.* **21**, 1263–1284 (2009).

[52] Cervera-Negueruela, M. et al. Smartwatch adherence patterns in a two-year longitudinal dataset in Huntington’s Disease. in *ADDS Basel 2025* (2025). Retrieved from https://medically.gene.com/global/en/unrestricted/neuroscience/ADDS-2025/adds-2025-poster-lugrin-smartwatch-adherence-patterns-i.html.

[53] Group, T. E. EuroQol - a new facility for the measurement of health-related quality of life. *Health Polic* **16**, 199–208 (1990). 10.1016/0168-8510(90)90421-9 10109801

[54] Walker, F. O. Huntington’s disease. *Lancet* **369**, 218–228 (2007). 17240289 10.1016/S0140-6736(07)60111-1

[55] Bachoud-Lévi, A.-C. et al. International Guidelines for the Treatment of Huntington’s Disease. *Front. Neurol.* **10**, 710 (2019). 31333565 10.3389/fneur.2019.00710 PMC6618900

[56] Fenney, A., Jog, M. S. & Duval, C. Short-term variability in amplitude and motor topography of whole-body involuntary movements in Parkinson’s disease dyskinesias and in Huntington’s chorea. *Clin. Neurol. Neurosurg.* **110**, 160–167 (2008). 18063471 10.1016/j.clineuro.2007.10.010

[57] Ding, C. & Peng, G. H. Minimum redundancy feature selection from microarray gene expression data. *J. Bioinform. Comput. Biol.* **3**, 185–205 (2005). 15852500 10.1142/s0219720005001004

[58] Cheng, W.-Y. et al. Human Activity Recognition from Sensor-Based Large-Scale Continuous Monitoring of Parkinson’s Disease Patients. *2017 IEEE/ACM Int. Conf. Connect. Health: Appl., Syst. Eng. Technol. (CHASE)* 249–250 10.1109/chase.2017.87 (2017).

[59] Hees, V. T. van et al. Separating Movement and Gravity Components in an Acceleration Signal and Implications for the Assessment of Human Daily Physical Activity. *PLoS ONE* **8**, e61691 (2013). 23626718 10.1371/journal.pone.0061691 PMC3634007

[60] Patil, P. & Parmigiani, G. Training replicable predictors in multiple studies. *Proc. Natl. Acad. Sci.* **115**, 2578–2583 (2018). 29531060 10.1073/pnas.1708283115 PMC5856504

