# Supplementary material for: Longitudinal assessment of chorea in Huntington’s disease using digital passive monitoring
Source: NPJ Digit Med. 2026 Apr 25;9:495. doi: 10.1038/s41746-026-02661-y (PMC13319451; doi:10.1038/s41746-026-02661-y)
Supplement: Supplementary file 1 — Supplementary Information [file 41746_2026_2661_MOESM1_ESM.pdf]

Supplementary material

Longitudinal assessment of chorea in Huntington's disease using digital passive monitoring

Claire Lugrin[1], Lidia Alecci[2], Cedric Simillion[1], Ekaterina Volkova-Volkmar[1], Louis-Solal Giboin[1], Fiona C. Kinsella[3], Peter McColgan[4], Edward J. Wild[3], Jonas Dorn[1*]

[1] Computational Sciences Center of Excellence, F. Hoffmann-La Roche Ltd, 4070 Basel, Switzerland
[2] Università della Svizzera italiana (USI), Lugano, Switzerland
[3] Huntington's Disease Centre, UCL Queen Square Institute of Neurology, University College London, London, United Kingdom
[4] Roche Products Ltd, Welwyn Garden City, United Kingdom
* Corresponding author: jonas.dorn@roche.com

Supplementary Tables

Supplementary Table 1: Inclusion criteria for the four different studies. *GENERATION HD1 original protocol (OP) and final protocol have the same inclusion criteria.*

	**HD Natural History Study**	**Digital-HD**			**GENERATION HD1**
		**People with clinically diagnosed HD**	**Gene-positive People pre-clinial motor diagnosis**	**Gene-negative control volunteers**	
Inclusion criteria	• Diagnosis of early manifest HD<br>• Shoulson-Fahn stage I/II disease<br>• UHDRS-TFC score 7-13)	• DCL = 4<br>• Stage I-III (UHDRS-TFC 4-13)<br>• CAG expansion ≥36<br>• Age 18-75 years	• DCL <4<br>• CAG expansion ≥40	• No known family history of HD or CAG expansion <36	• Diagnosis of manifest HD<br>• DCL = 4<br>• Independence scale score ≥70<br>• CAP score >400

Exclusion criteria	• Any serious medical condition of laboratory finding that, in the investigator's judgment, precludes the safe participation in and completion of the study<br>• Pregnancy, breastfeeding, or intention of becoming pregnant during the study<br>• Current or previous use of an antisense oligonucleotide (including small interfering RNA)<br>• Current use of antipsychotics prescribed for psychosis, cholinesterase inhibitors, memantine, amantadine, or riluzole including use within 12 weeks of enrollment<br>• Treatment with an investigational drug within 30 days prior to screening or 5 half-lives of the investigational drug, whichever is longer	• Any serious medical condition of laboratory finding that, in the investigator's judgment, precludes the safe participation in and completion of the study<br>• Inability or unwillingness to undertake any of the essential study procedures<br>• Current use of investigational drug or participation in a clinical drug trial within 30 days prior to sampling visit<br>• Current intoxication, or drug or alcohol abuse/dependence<br>• Use of inappropriate or unstable dose of any antidepressant, psychoactive, psychotropic or other medication, or nutraceuticals used to treat HD	• Any serious medical condition of laboratory finding that, in the investigator's judgment, precludes the safe participation in and completion of the study<br>• Pregnancy, breastfeeding, or intention of becoming pregnant during the study or within 5 months after the final dose of the study drug<br>• Current or previous use of an antisense oligonucleotide (including small interfering RNA)<br>• Current use of antipsychotics prescribed for psychosis, cholinesterase inhibitors, memantine, amantadine, or riluzole including use within 12 weeks of enrollment Treatment with an investigational drug within 30 days prior to screening or 5 half-lives of the investigational drug, whichever is longer

[a] At enrollment in the phase 1/2a study.
CAG, cytosine-adenine-guanine; CAP, CAG-Age-Product; DCL, diagnostic confidence level; HD, Huntington's Disease; UHDRS-TFC, Unified Huntington's Disease Rating Scale-Total Functional Capacity.

Supplementary Table 2: Demographics and baseline characteristics of the participants. *Mean and standard deviation of demographic and clinical scores of the participants of the different studies during the baseline assessment. F: Female, UHDRS: Unified Huntington's Disease Rating Scale, TFC: Total Functional Capacity subscale of the UHDRS, TMS: Total Motor Score subscale of the UHDRS.*

	**N**	**Age**	**Sex**	**CAG repeats**	**cUHDRS (baseline)**	**TFC (baseline)**	**TMS (baseline)**	**In-clinic upper limb chorea (baseline)**
Digital-HD After CMD	40	55.7 (11.0)	F: 19 (47.5%)	42.6 (3.3)	10.4 (3.8)	10.7 (2.2)	32.4 (17.0)	1.8 (0.7)
Digital-HD Before CMD	40	42.6 (9.1)	F: 22 (55.0%)	41.9 (1.8)	17.4 (1.9)	12.9 (0.2)	4.5 (3.3)	0.6 (0.5)
Digital-HD Gene-negative control	40	43.9 (14.1)	F: 18 (45.0%)	-	18.0 (1.4)	13.0 (0.0)	0.9 (1.9)	0.2 (0.4)
GENERATION HD1 OP	107	46.0 (10.2)	F: 61 (57.0%)	45.3 (3.2)	11.7 (2.7)	10.6 (2.2)	27.3 (13.2)	1.4 (0.7)
NHS	95	48.1 (9.9)	F: 36 (37.9%)	44.2 (3.1)	12.7 (2.4)	11.0 (1.5)	22.1 (10.9)	1.2 (0.7)
GENERATION HD1	786	48.1 (9.6)	F: 356 (45.0%)	44.9 (3.2)	11.4 (2.6)	10.4 (2.2)	28.4 (12.9)	1.4 (0.8)

Supplementary Table 3: Summary of the top 22 features selected for chorea estimation. *Features are ranked by their maximum quality metric (cross sectional correlation with in-clinic chorea times intra-class correlation coefficient, see Supplementary Methods and Supplementary Figures 2-4), and lowest maximum correlation with already selected features (Supplementary Methods, Supplementary Figure 5). The final feature set characterizes four primary signal domains: complexity features quantifying the unpredictability of choreic jerks (Lyapunov exponent, Sample Entropy), magnitude features capturing hyperkinetic displacement (Path Length, ENMO, Accelerometer Magnitude, Path Kurtosis), stability features measuring signal fluctuations and postural control (Base Line Crossings, Orientation Stability, Derivatives), and frequency features distinguishing choreic spectral signatures from voluntary motion (Power 50th percentile, Frequency Dispersion). To ensure phenotypic stability, features were derived through a hierarchical temporal aggregation where raw signal was first processed within active movement bouts, then aggregated to represent daily distributional characteristics, and finally averaged over a two-week period to provide a robust objective proxy. ENMO: Euclidean Norm Minus One, MAD: Mean Absolute Deviation, SD: Standard Deviation.*

**Rank**	**Feature**	**Quality metric**	**Maximum correlation**
1	Median of 10th percentile Max Lyapunov Exponent	0.57	0.00
2	30th percentile of 10th percentile Path Length	0.54	0.47
3	Median of 10th percentile Sample Entropy	0.52	0.44
4	90th percentile of Peak Sample Entropy	0.45	0.41
5	40th percentile of Mode Power 50th percentile	0.47	0.53
6	60th percentile of MAD Power 50th percentile	0.46	0.52
7	90th percentile of SD Sample Entropy	0.49	0.61
8	Skewness of Mode Base Line Crossings	0.45	0.56
9	Mean of MAD Orientation Stability	0.41	0.49
10	Skewness of Bout Derivative 40th percentile	0.40	0.44
11	Daily SD of Derivative 60th percentile	0.44	0.58
12	70th percentile of Mean Path Kurtosis	0.40	0.49
13	SD of SD Path Length	0.38	0.37
14	20th percentile of MAD Base Line Crossings	0.39	0.49
15	10th percentile of Mode Sample Entropy	0.45	0.61
16	Mean of Max Frequency Dispersion	0.38	0.43
17	Daily 90th percentile of Derivative 40th percentile	0.39	0.51
18	SD of Mode Sample Entropy	0.43	0.60
19	70th percentile of Mode Magnitude SD	0.39	0.53

20	20th percentile of MAD Frequency Dispersion	0.44	0.63
21	Mode of movement bout ENMO SD	0.35	0.39
22	10th percentile of Mode Max Lyapunov Exponent	0.46	0.65

Supplementary Table 4: Correlations between the DPCS and in-clinic chorea at different visits to the clinic. *Spearman correlations between the DPCS and in-clinic chorea for the held-out participants (N = 310) at different visits to the clinic. The correlation coefficient (rho), p-value, and number of participants with both passive-monitoring and clinical assessments (n) are reported.*

**Visit**	**rho**	**p value**	**N**
baseline	0.453	< 0.001	276
Week 4	0.401	< 0.001	262
Week 16	0.435	< 0.001	227
Week 38	0.446	< 0.001	166
Week 53	0.354	< 0.001	120
Week 62	0.492	< 0.001	91
Week 85	0.341	0.011	55
mean	0.417		

Supplementary Table 5: Mixed-effect regression of time on DPCS change-from-baseline for the placebo arm of GENERATION HD1 (longitudinal test and held-out participants) . *Fixed-effect coefficients (Estimates), standard error, z-value, p-value and confidence interval (CI) of linear regressions of time (in years) on DPCS change-from-baseline, including a random intercept and random slope of for each participant, and sex, age, cag repeats and baseline DPCS as control variables.*

**Parameter**	**Estimate**	**Ste**	**z-value**	**P>|z|**	**CI low**	**CI high**
Intercept	-0.228	0.509	-0.448	0.654	-1.226	0.77
sex	0.08	0.033	2.429	0.015	0.015	0.144
symptomatic treatment	-0.007	0.032	-0.209	0.835	-0.069	0.056
year	0.126	0.025	5.097	< 0.001	0.077	0.174
age	0.003	0.003	0.939	0.347	-0.003	0.009
cag repeats	0.007	0.009	0.793	0.428	-0.01	0.024
baseline	-0.199	0.034	-5.782	< 0.001	-0.267	-0.132

Supplementary Table 6: Mixed-effect regression of time on DPCS change-from-baseline for the participants of NHS (train set). *Fixed-effect coefficients (Estimates), standard error, z-value, p-value and confidence interval (CI) of linear regressions of time (in years) on DPCS change-from-baseline, including a random intercept and random slope of for each participant, and sex, age, cag repeats and baseline DPCS as control variables.*

Parameter	Estimate	Ste	z-value	P>|z|	CI low	CI high
Intercept	1.043	0.932	1.12	0.263	-0.783	2.869
sex	-0.054	0.054	-1.006	0.315	-0.159	0.051
symptomatic treatment	0.093	0.06	1.554	0.12	-0.024	0.211
year	0.149	0.04	3.671	< 0.001	0.069	0.228
age	-0.007	0.005	-1.397	0.162	-0.017	0.003
cag repeats	-0.012	0.016	-0.749	0.454	-0.044	0.02
baseline	-0.113	0.055	-2.054	0.04	-0.221	-0.005

Supplementary Table 7: Mixed-effect regression of time on DPCS change-from-baseline for the participants of the GENERATION HD1 placebo arm and NHS studies *Fixed-effect coefficients (Estimates), standard error, z-value, p-value and confidence interval (CI) of linear regressions of time (in years), study, and their interaction on DPCS change-from-baseline, including a random intercept and random slope of for each participant, and sex, age, cag repeats and baseline DPCS as control variables.*

Parameter	Estimate	Ste	z-value	P>|z|	CI low	CI high
Intercept	0.016	0.418	0.038	0.97	-0.804	0.835
study	-0.093	0.078	-1.193	0.233	-0.246	0.06
sex	0.046	0.026	1.757	0.079	-0.005	0.096
year	0.12	0.023	5.201	0	0.075	0.166
year : study	0.024	0.047	0.521	0.603	-0.067	0.116
Age	0.001	0.002	0.245	0.806	-0.004	0.005
cag repeats	0.005	0.007	0.671	0.502	-0.009	0.019
baseline	-0.214	0.03	-7.151	0	-0.273	-0.155
baseline : study	0.067	0.06	1.119	0.263	-0.05	0.184

Supplementary Table 8: List of symptomatic treatments prescribed against chorea and included as a dummy control variable. *Molecule names and number of participants reporting having taken this medication on at least one clinical visit during the NHS and GENERATION HD1 studies. Medications indicated as sedative for MRI or lumbar puncture were excluded from the analysis.*

Treatment	Number of participants
ALPRAZOLAM; SULPIRIDE	1
AMANTADINE	10
AMANTADINE HYDROCHLORIDE	2
AMISULPRIDE	1
ARIPIPRAZOLE	41
BROMAZEPAM	2
CLONAZEPAM	57
DELORAZEPAM	1
DEUTETRABENAZINE	178
DIAZEPAM	20
LORAZEPAM	64
LORMETAZEPAM	4
NORDAZEPAM	2
OLANZAPINE	190
OXAZEPAM	4
QUETIAPINE	43
QUETIAPINE FUMARATE	49
RISPERIDONE	164
SULPIRIDE	5
TEMAZEPAM	3
TETRABENAZINE	270

Supplementary Table 9: Mixed-effect regression of time on DPCS change-from-baseline for the participants of the GENERATION HD1 placebo arm and NHS studies, including symptomatic treatments. *Fixed-effect coefficients (Estimates), standard error, z-value, p-value and confidence interval (CI) of linear regressions of time (in years), study, and their interaction on DPCS change-from-baseline, including a random intercept and random slope of for each participant, and symptomatic treatments, sex, age, cag repeats and baseline DPCS as control variables. The regressor symptomatic treatment is a dummy variable taking value 1 when participants received treatment against chorea at any time point during the studies, and value 0 otherwise (see Supplementary Table 2 for a list of treatments considered). This variable accounts for effects of symptomatic treatments on the progression of chorea. Note that the coefficient associated with that variable cannot be used to make inference on potential treatment efficacy of concomitant medication, as the study participants have not been randomized to these treatments, and any comparison would most likely include substantial bias. A Likelihood Ratio Test showed no significant difference between the models with or without symptomatic treatments (likelihood ratio statistic: -5.44, p = 1).*

Parameter	Estimate	Ste	z-value	P>|z|	CI low	CI high
Intercept	0.009	0.423	0.021	0.983	-0.819	0.837
study	-0.093	0.078	-1.189	0.235	-0.246	0.06
sex	0.046	0.026	1.757	0.079	-0.005	0.097
symptomatic treatment	-0.003	0.026	-0.117	0.907	-0.054	0.048
year	0.12	0.023	5.202	< 0.001	0.075	0.166
year : study	0.024	0.047	0.52	0.603	-0.067	0.116
age	0.001	0.002	0.261	0.794	-0.004	0.005
cag repeats	0.005	0.007	0.681	0.496	-0.009	0.019
baseline	-0.214	0.03	-7.132	< 0.001	-0.273	-0.155
baseline : study	0.066	0.06	1.107	0.268	-0.051	0.184

Supplementary Table 10: List of the approving IRBs (Institutional Review Boards) and ECs (Ethics Committee) for the GENERATION HD1 study.

IRB / EC	Country
London – West London & GTAC	United Kingdom
Comité de Ética en Investigación Fundación Ineba	Argentina
CEIC Hospital Vall d'Hebron	Spain
Békés Megyei Képviselő Testület Pándy Kálmán Kórház Kutatásetikai Bizottsága	Hungary
Komisja Bioetyczna przy Okręgowej Izbie Lekarskiej w Gdańsku	Poland
Comité de Bioética de Investigación Hospital General de Agudos J.M. Ramos Mejía	Argentina
Ethik-Kommission der Ärztekammer Westfalen-Lippe und der Medizinischen Fakultät der WWU Münster	Germany
Ethik-Kommission der Medizinischen Fakultät der Ruhr-Universität Bochum	Germany
Ethik-Kommission Berlin – Landesamt für Gesundheit und Soziales	Germany
Ethik-Kommission der Universität Ulm	Germany
Ethik-Kommission an der Medizinischen Fakultät der Uniklinik Aachen	Germany
Ethik-Kommission der Medizinischen Fakultät der Friedrich-Alexander-Universität Erlangen-Nürnberg	Germany
Western Sydney Local Health District Research Governance Office	Australia
Kantonale Ethikkommission Bern (KEK)	Switzerland
De Videnskabsetiske Komitéer for Region Hovedstaden	Denmark
Central Committee on Research Involving Human Subjects (CCMO)	Netherlands
WIRB – Western Institutional Review Board	United States
Advarra	United States
The Committee for the Protection of Human Subjects	United States
Georgetown-Howard Universities Center for Clinical and Translational Science IRB	United States
UPMC Office of Sponsored Programs and Research Support (OSPARS)	United States
Johns Hopkins Medicine Institutional Review Board	United States
Columbia University Medical Center IRB	United States
UBC Clinical Research Ethics Board	Canada

IRB / EC	Country
Comité d’éthique de la recherche, CHUM	Canada
Institutional Review Board – UC Davis Medical Center	United States
Washington University School of Medicine, Human Research Protection Office	United States
UCSD Human Research Protections Program / Altman Clinical and Translational Institute	United States
Committee on Clinical Investigations	United States
CPP Ouest VI, Centre Hospitalier Universitaire Cavale Blanche	France
Ethikkommission der Medizinischen Fakultät der Universität Innsbruck	Austria
EC at Research Center of Neurology; Ethics Committee	Russia
Ethikkommission des Land Salzburg	Austria
Comitato Etico della Fondazione IRCCS Istituto Neurologico Carlo Besta di Milano	Italy
Comitato Etico dell’IRCCS Giovanni Paolo II di Bari presso IRCCS Casa Sollievo della Sofferenza	Italy
Comitato Etico Interaziendale Bologna-Imola-Ferrara	Italy
Health and Disability Ethics Committees, Ministry of Health	New Zealand
Comité de Revisión Hospital Británico	Argentina
St. Joseph’s Hospital & Medical Center IRB	United States
University of Utah IRB	United States
Ottawa Health Science Network Research Ethics Board (OHSNREB)	Canada
Nova Scotia Health Authority Research Ethics Board	Canada
Health Research Ethics Board, University of Alberta	Canada
Central HDEC, Ministry of Health	New Zealand
Ethikkommission der Universität zu Lübeck	Germany
Comitato Etico A.O. Careggi / Comitato Etico Area Vasta Centro	Italy
IRB/IEC at FSBI Federal Siberian Scientific and Clinical Center (FMBA)	Russia
Comitato Etico Regione Liguria (Sezione 2)	Italy
Comitato Etico dell’Università “Sapienza”	Italy
Vanderbilt University Institutional Review Board	United States
Ethikkommission Nordwest- und Zentralschweiz (EKNZ)	Switzerland

IRB / EC	Country
Ethics Committee at Vashe Zdorovie	Russia
Ethikkommission an der Medizinischen Fakultät der Rheinischen Friedrich-Wilhelms-Universität Bonn	Germany
Comité de Ética Servicio de Salud Metropolitano Norte	Chile
National Hospital Organization Sagamihara National Hospital IRB	Japan
NHO Niigata Hospital IRB	Japan
Osaka General Medical Center IRB	Japan
National Center of Neurology and Psychiatry IRB	Japan
Kuwana City Medical Center IRB	Japan
Okayama University Hospital IRB	Japan

Supplementary Table 11: List of the approving IRBs and ECs for the NHS study.

IRB / Ethics Committee Name	Country
Ethik-Kommission der Universität Ulm	Germany
Ethik-Kommission der Medizinischen Fakultät der Ruhr-Universität Bochum	Germany
London – Camden & Kings Cross Research Ethics Committee	United Kingdom
Johns Hopkins Medicine Institutional Review Board	United States
Columbia University Medical Center (CUMC) IRB	United States
Advarra	United States
MedStar Health Research Institute – Georgetown University Oncology IRB	United States
University of British Columbia Clinical Research Ethics Board	Canada
HCA-HealthONE	United States
Advarra Inc.	Canada

Supplementary Figures

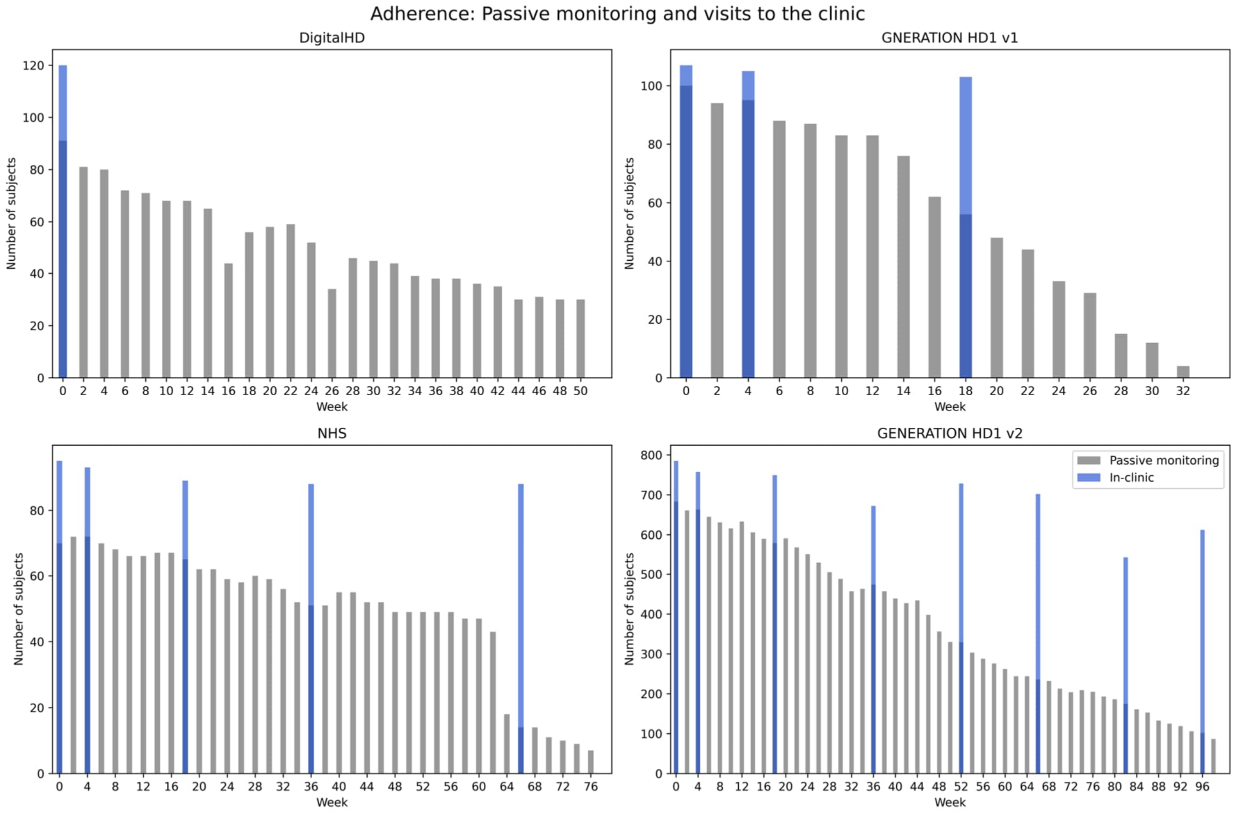

Supplementary Figure 1: Passive monitoring and in-clinic adherence for the different studies. *The grey bars represent the number of participants collecting sufficient passive-monitoring data for the Digital Passive-monitoring Chorea Score (DPCS) to be estimated for each two-week period following the baseline visit of the studies. The blue bars represent the number of participants for which in-clinic assessments were recorded at each planned visit.*

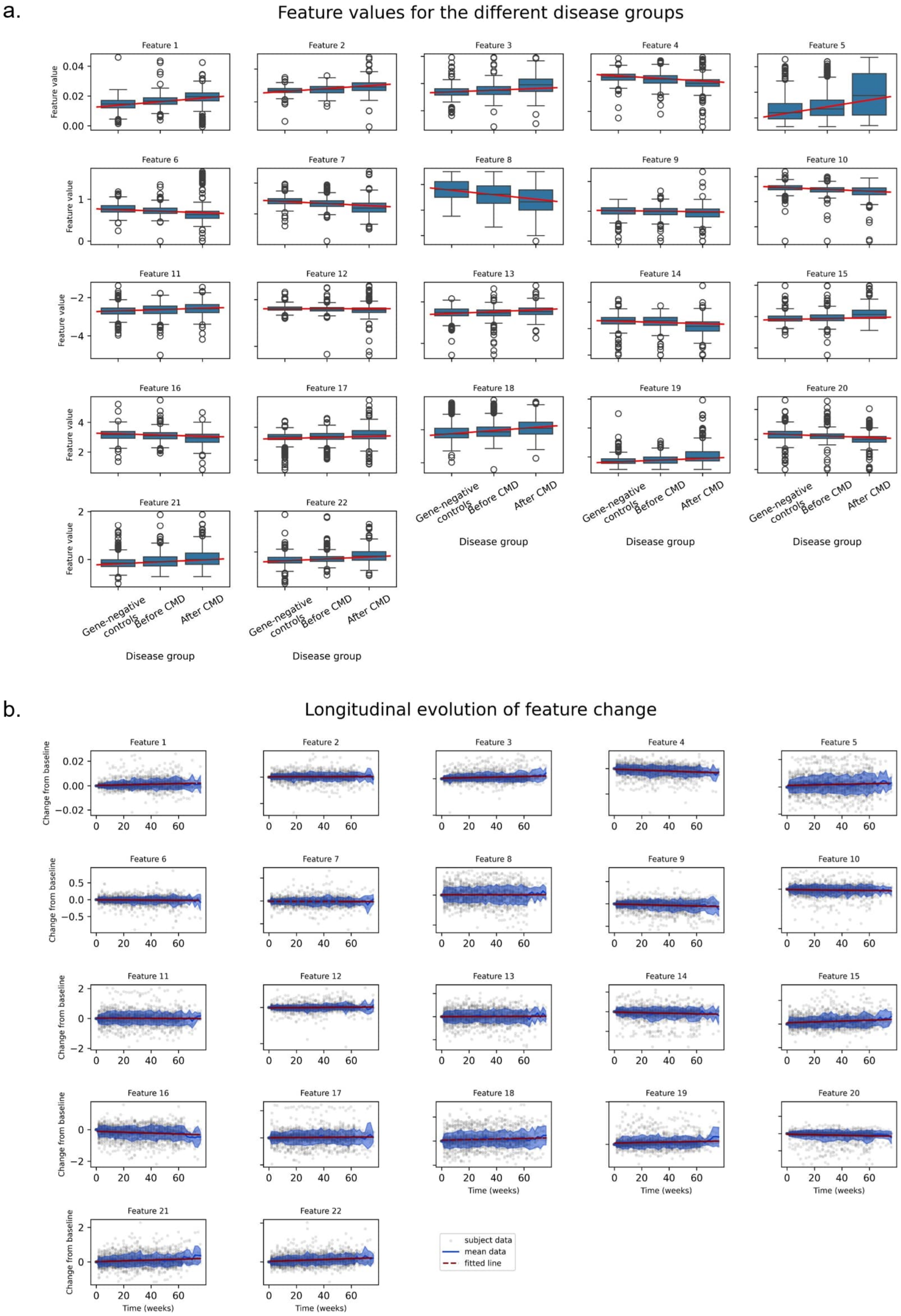

Supplementary Figure 2: Disease group and longitudinal progression slopes used to exclude features with incongruent patterns. ***a.*** *Values of the 16 top-ranking features for the different groups of Digital-HD (control participants, participants carrying the mutation associated with HD but before clinical motor diagnostics (CMD), and participants diagnosed with HD (after*

CMD). Boxplots represent the median and quartiles of feature values across all participants of a group. The red lines represent regressions between feature values and disease groups. The slope of this regression line is used in the congruent validity step. ***b.*** *Evolution of change-from-baseline of the 16 top ranked features for NHS. Each dot represents a participant, the blue line and shaded area represent the mean and standard deviation across participants, and the red line a regression between week in study and feature values. The slope of the regression line is used in the congruent validity step.*

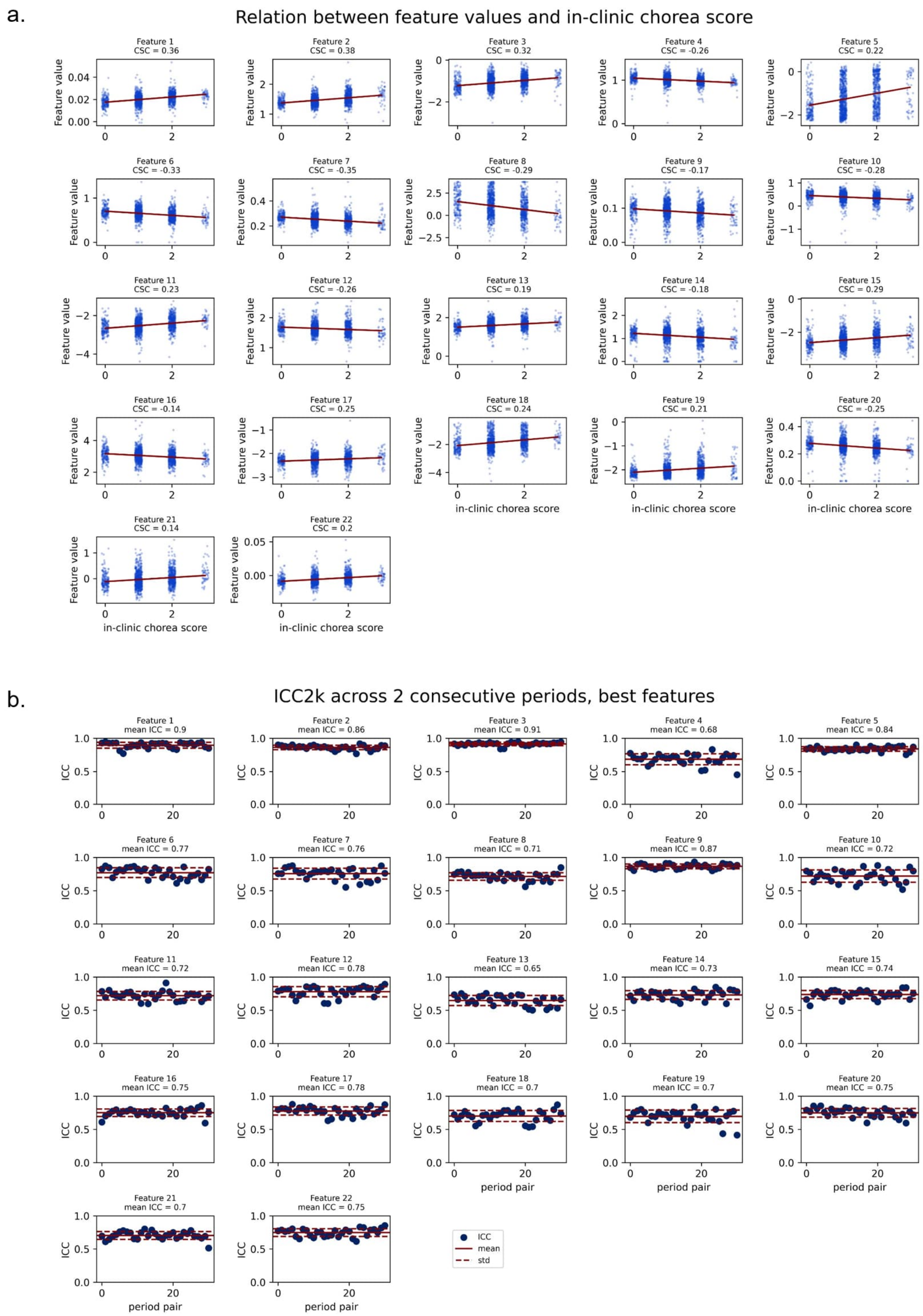

Supplementary Figure 3: Cross-Sectional Correlation (CSC) and Intraclass Correlation Coefficient (ICC) used to define the feature quality metric. a. *Relation between the in-clinic chorea score and value of the top-ranking features. Each dot represents one visit to the clinic of one participant, and the red line illustrates a regression between the feature value and the in-clinic chorea. The reported CSC is the average Spearman correlation coefficient computed for the different visits to the clinic (until week 38) and averaged across visits for the NHS and*

GENERATION HD1 studies. ***b.*** *ICC computed for each consecutive pairs of two-week periods for the top-ranking features. Each dot represents a period pair, the red lines represent the mean ICC and standard deviation across periods. The mean ICC across time is used as a component of the feature quality metric.*

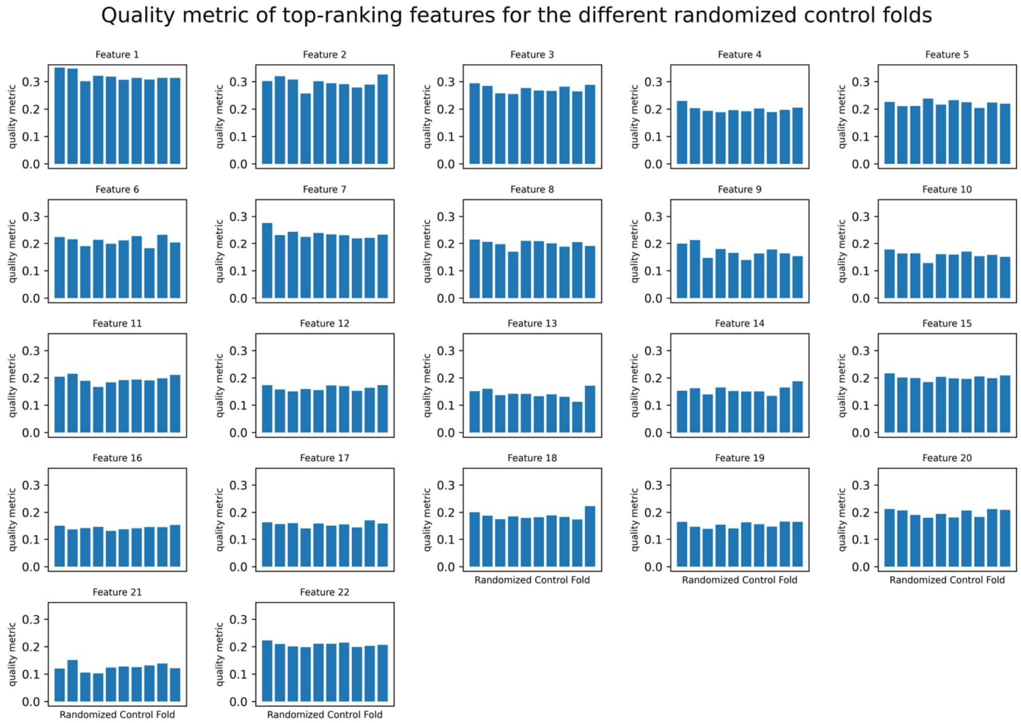

Supplementary Figure 4: Feature quality metric across randomized control folds. *The feature quality was measured as abs(CSC) * ICC and computed for 10 random folds composed of 80% of the training participants. Only features with a mean / standard deviation of the quality metric above 2 were considered for ranking. The bars represent the quality metric of the top-ranking features for each randomized control folds, showing the stability of the metric.*

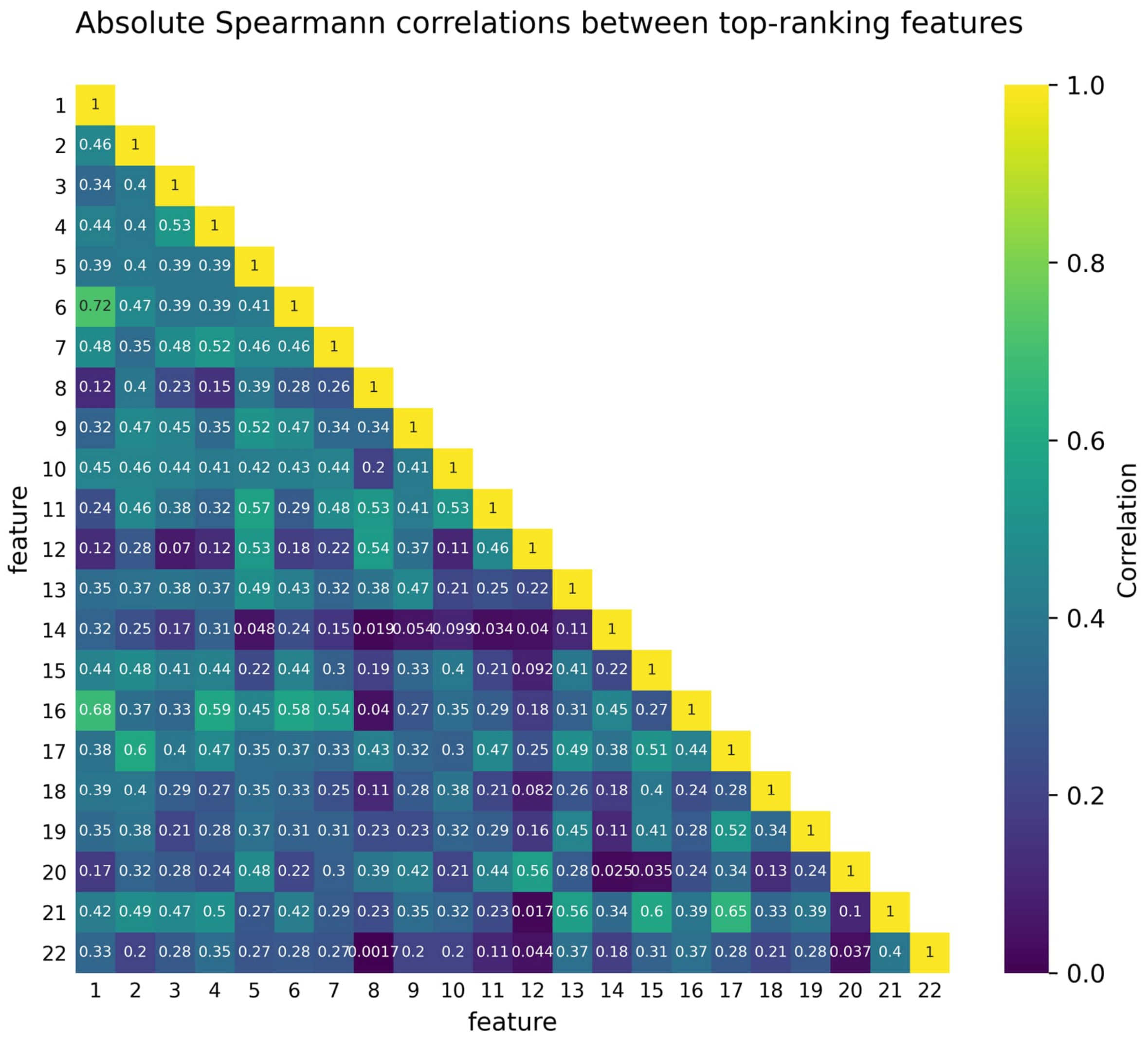

Supplementary Figure 5: Maximum Relevance Minimum Redundancy ranking algorithm provides low-correlated top features. *Correlation matrix of the 22 top-ranked features. The colors and numbers represent Spearman correlation coefficients (absolute value) , rows and columns are the different features.*

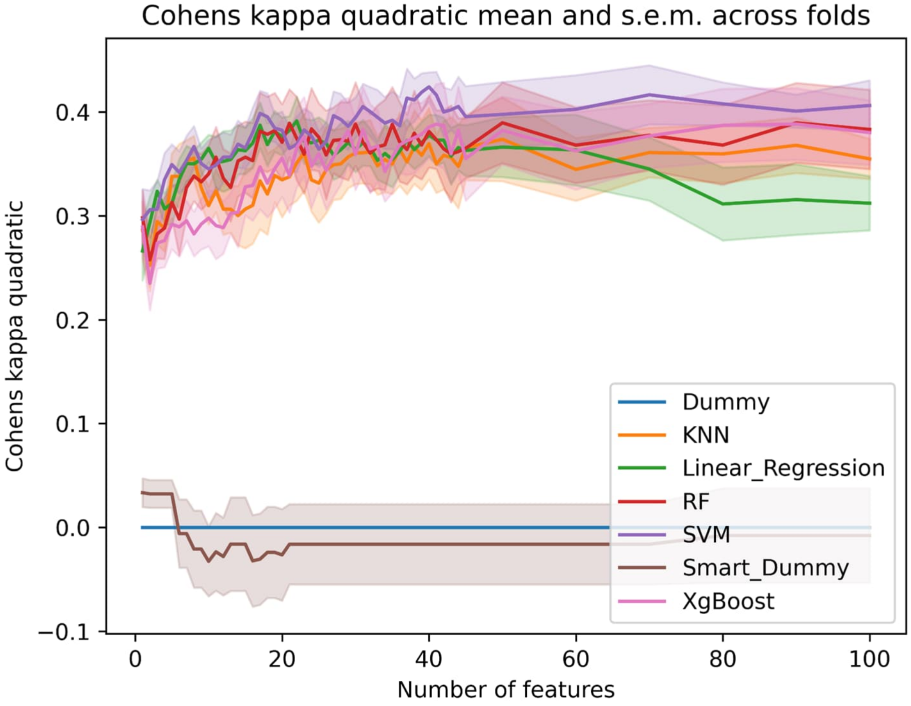

Supplementary Figure 6: Model performance and differences across cross-validation folds. *Evolution of different model performance when features are iteratively added (1 to 100 features, ranked by quality metric using MRMR). Model performance is defined as the mean of the quadratic weighted Cohen's kappa across the 6 outer cross-validation test sets (i.e. 6 iterations of training and testing models on different data sets) and represented as plain lines. Shaded areas show the standard deviation across the cross-validation folds.*

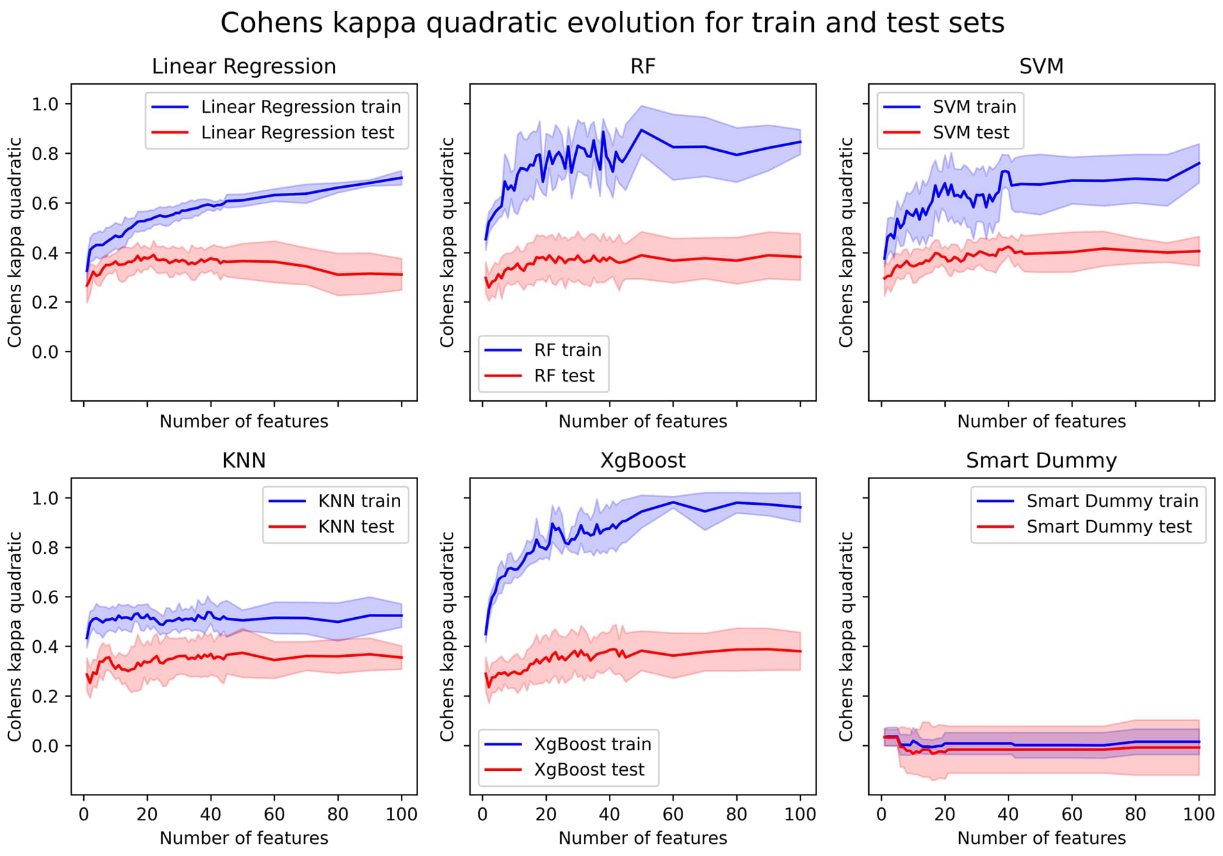

Supplementary Figure 7: Comparison of model performances on training and testing sets across 6 outer cross-validation folds. *Different models were trained using sequentially added top-raking features. The models were then applied to the unseen test-set and to the sets used for training them (train sets). The lines represent the average Cohen's kappa across folds for the train (blue) and test (red) sets, and the shaded areas represent the standard deviations. SVM (Support Vector Machine), XGBoost and RF (Random Forrest) models are overfitting the training data: They perform better on the data they have been trained on compared to test data. Linear Regression is only moderately over-performing on the training set when up to 22 features are added, making it a better candidate for generalizing to new data.*

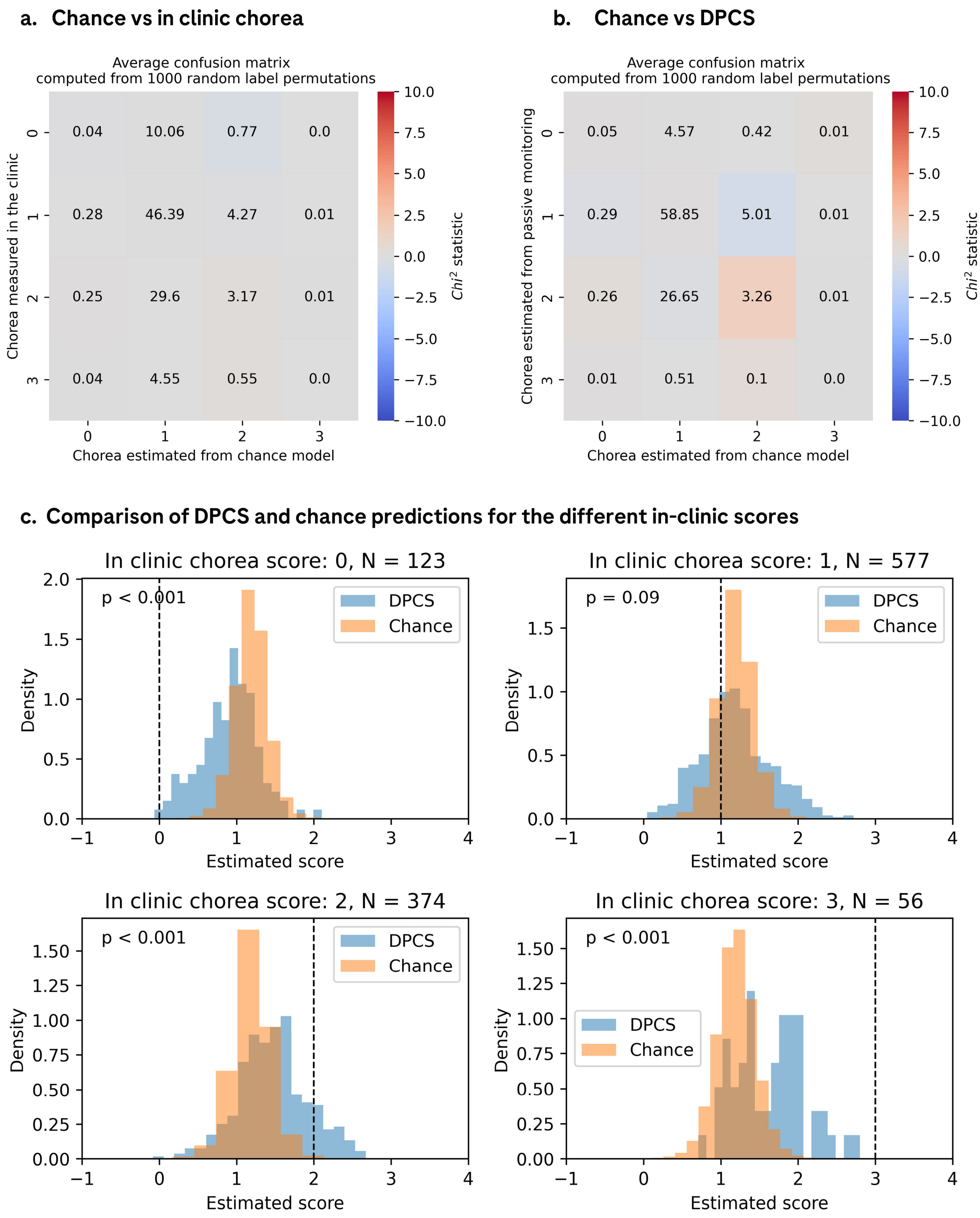

Supplementary Figure 8: Label permutations show that the model performs better than chance, but underestimate high chorea values and overestimates low values. a and b. *Average confusion matrix across 1000 repetitions of model training using random permutations of labels against in clinic chorea (**a**), or DPCS (**b**).The numbers represent the average number of participants in each category (estimations from the random model on the x axis vs in-clinic chorea (a) or DPCS (b) on the y axis), and the colors the Chi2 statistics, reflecting the deviation from the expected number of participants in a category if the in-clinic and passive monitoring chorea scores*

were drawn from independent distributions: Observation more (resp. less) frequent than expected under the independence hypothesis are represented in red (res. blue), grey colors show that the distribution of chance model predictions are independent from the in-clinic chorea (a) and DPCS (b) distributions. ***c.*** *Distributions of scores estimated from the linear regression 22 features (DPCS in blue) and estimated from 1000 models trained on random permutations of labels (chance in orange) for the four values of in-clinic chorea (dotted lines). Chance estimates all scores around 1, while the DPCS provides estimates closer to in-clinic scores, but underestimates scores of 3 and overestimates scores of 0.*

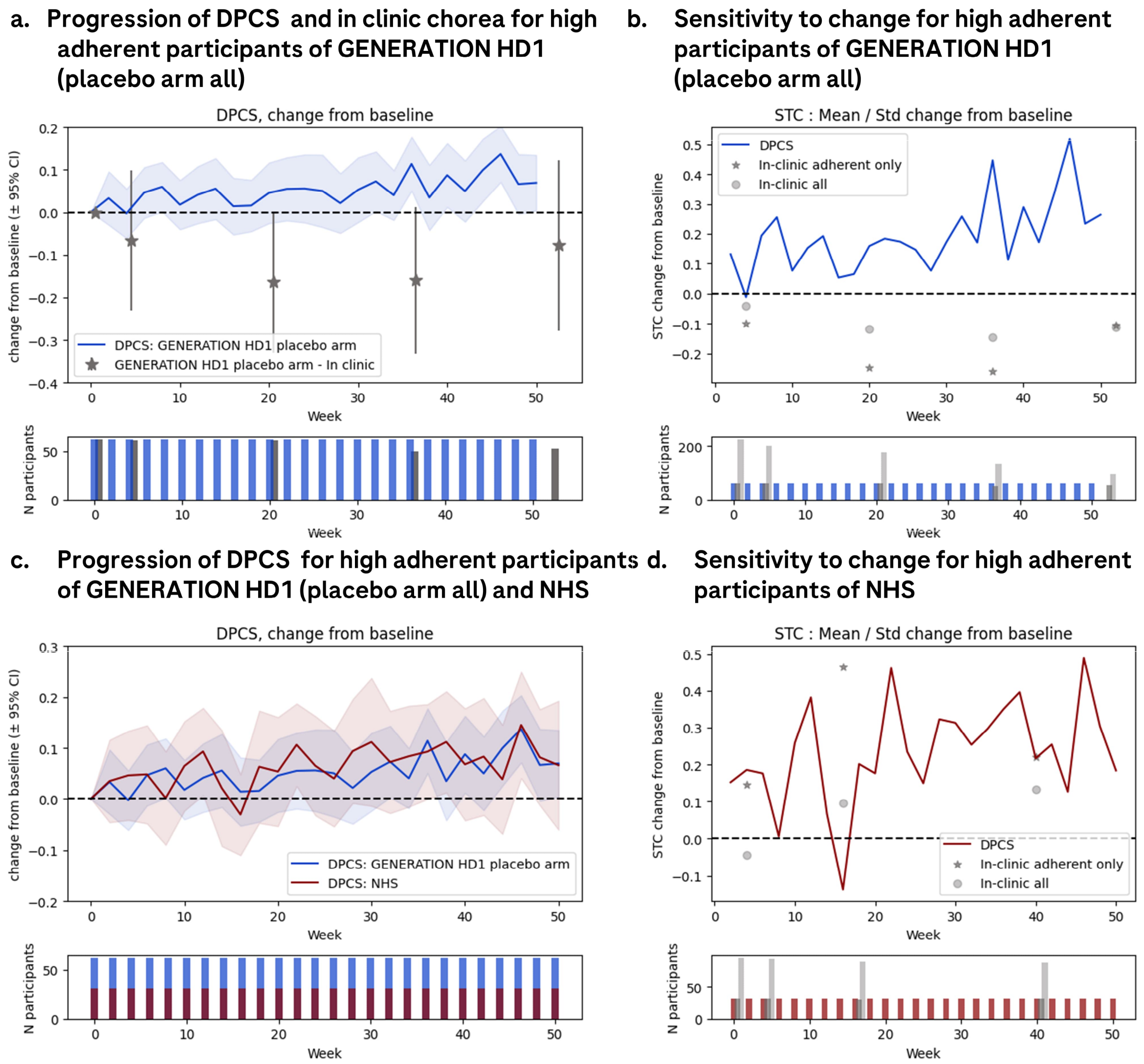

Supplementary Figure 9: Progression of DPCS and sensitivity to change for participants with a consistent high adherence. *Replicates Figure 4a, e and f. Only participants who collected passive monitoring data at all time points between baseline and week 50 were included in the current analysis.* ***a and c.*** *Change-from-baseline of in-clinic (grey) and DPCS (GENERATION HD: blue, NHS: red) over the course of the studies. The colored line and shaded area represent the average and 95% confidence interval of the DPCS across all participants collecting passive monitoring data, and the grey stars and lines represent the average in-clinic chorea score and 95% confidence interval of the same participants. The bottom plot shows the number of participants who collected passive monitoring data and were therefore included in the top plot.* ***b and d.*** *Sensitivity to change: Mean / standard deviation of change from baseline across participants collecting passive monitoring data. The colored lines represent the DPCS of participants collecting data at all time points, the stars represent the in-clinic chorea of the same participants, and the dots represent in-clinic chorea of all participants having collected in-clinic chorea. This shows a consistently high sensitivity to change, despite a smaller sample size.*

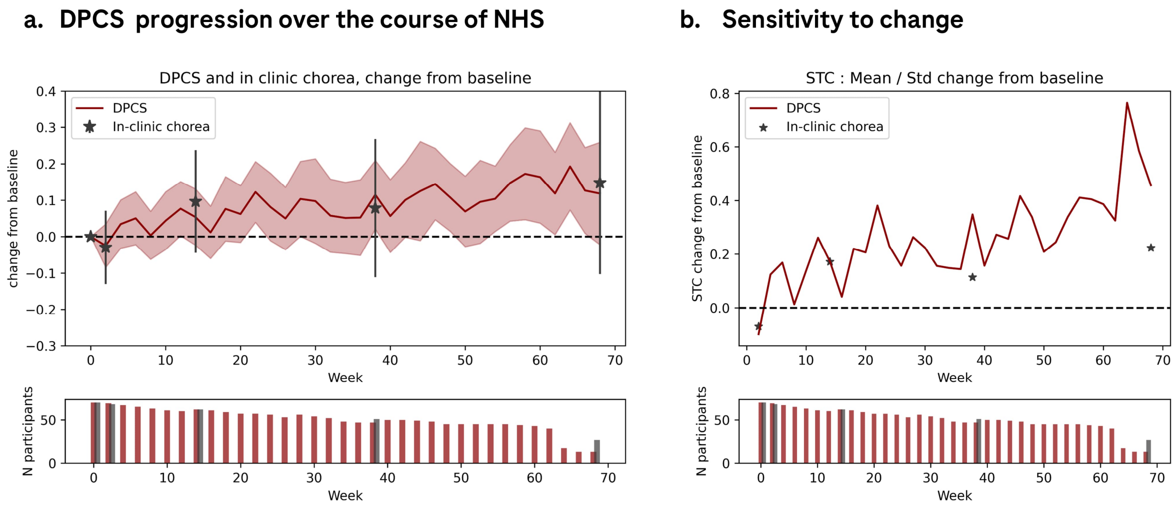

Supplementary Figure 10: Progression of DPCS over the course of NHS (N = 70). *The final model was applied to passive monitoring data, producing an estimate of chorea every two weeks.* ***a.*** *Change-from-baseline of in-clinic (grey) and DPCS (red) over the course of the NHS study. The red line and shaded area represent the average and 95% confidence interval of the DPCS across all participants collecting passive monitoring data, and the grey stars and lines represent the average in-clinic chorea score and 95% confidence interval of the same participants. The bottom plot shows the number of participants who collected passive monitoring data and were therefore included in the top plot.* ***b.*** *Sensitivity to change: Mean / standard deviation of change from baseline across all participants collecting passive monitoring data.*

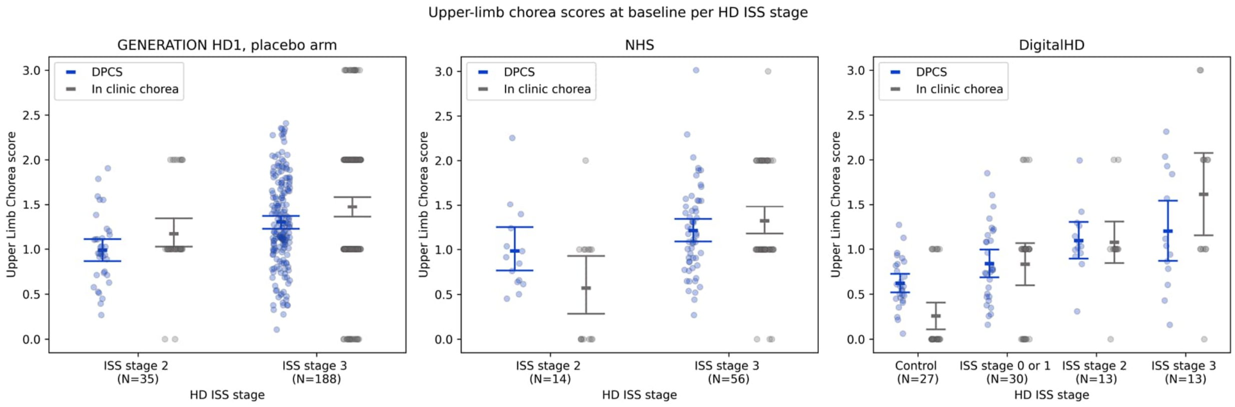

Supplementary Figure 11: DPCS and in clinic chorea at baseline for different HD-ISS stages. *DPCS (blue) and in-clinic chorea (grey) at baseline for the different retrospective HD-ISS staging of GENERATION HD placebo arm (all data including training and validation sets), NHS (training set) and Digital-HD (training set). Error bars represent 95% confidence intervals. HD-ISS stages were retrospectively computed for each participant based on the available data. The datasets were collected before the creation of the new staging system and do not contain the MRI information necessary to differentiate ISS stage 0 and stage 1.*
